# Tea polyphenol modified, photothermal responsive and ROS generative black phosphorus quantum dots as nanoplatforms for promoting MRSA infected wounds healing in diabetic rats

**DOI:** 10.1186/s12951-021-01106-w

**Published:** 2021-11-10

**Authors:** Shibo Xu, Linna Chang, Yanan Hu, Xingjun Zhao, Shuocheng Huang, Zhenhua Chen, Xiuli Ren, Xifan Mei

**Affiliations:** Jinzhou Medical University, Jinzhou, 121001 Liaoning China

**Keywords:** Multifunctional nanoplatforms, Black phosphorus quantum dots, Diabetic deep-burn wound healing, Photocatalytic, Photothermal

## Abstract

**Background:**

Healing of MRSA (methicillin-resistant Staphylococcus aureus) infected deep burn wounds (MIDBW) in diabetic patients remains an obstacle but is a cutting-edge research problem in clinical science. Surgical debridement and continuous antibiotic use remain the primary clinical treatment for MIDBW. However, suboptimal pharmacokinetics and high doses of antibiotics often cause serious side effects such as fatal complications of drug-resistant bacterial infections. MRSA, which causes wound infection, is currently a bacterium of concern in diabetic wound healing. In more severe cases, it can even lead to amputation of the patient's limb. The development of bioactive nanomaterials that can promote infected wound healing is significant.

**Results:**

The present work proposed a strategy of using EGCG (Epigallocatechin gallate) modified black phosphorus quantum dots (BPQDs) as therapeutic nanoplatforms for MIDBW to achieve the synergistic functions of NIR (near-infrared)-response, ROS-generation, sterilization, and promoting wound healing. The electron spin resonance results revealed that EGCG-BPQDs@H had a more vital photocatalytic ability to produce singlet oxygen than BPQDs@H. The inhibition results indicated an effective bactericidal rate of 88.6% against MRSA. Molecular biology analysis demonstrated that EGCG-BPQDs significantly upregulated CD31 nearly fourfold and basic fibroblast growth factor (bFGF) nearly twofold, which were beneficial for promoting the proliferation of vascular endothelial cells and skin epidermal cells. Under NIR irradiation, EGCG-BPQDs hydrogel (EGCG-BPQDs@H) treated MIDBW area could rapidly raise temperature up to 55 °C for sterilization. The MIBDW closure rate of rats after 21 days of treatment was 92.4%, much better than that of 61.1% of the control group. The engineered EGCG-BPQDs@H were found to promote MIDBW healing by triggering the PI3K/AKT and ERK1/2 signaling pathways, which could enhance cell proliferation and differentiation. In addition, intravenous circulation experiment showed good biocompatibility of EGCG-BPQDs@H. No significant damage to major organs was observed in rats.

**Conclusions:**

The obtained results demonstrated that EGCG-BPQDs@H achieved the synergistic functions of photocatalytic property, photothermal effects and promoted wound healing, and are promising multifunctional nanoplatforms for MIDBW healing in diabetics.

**Graphical Abstract:**

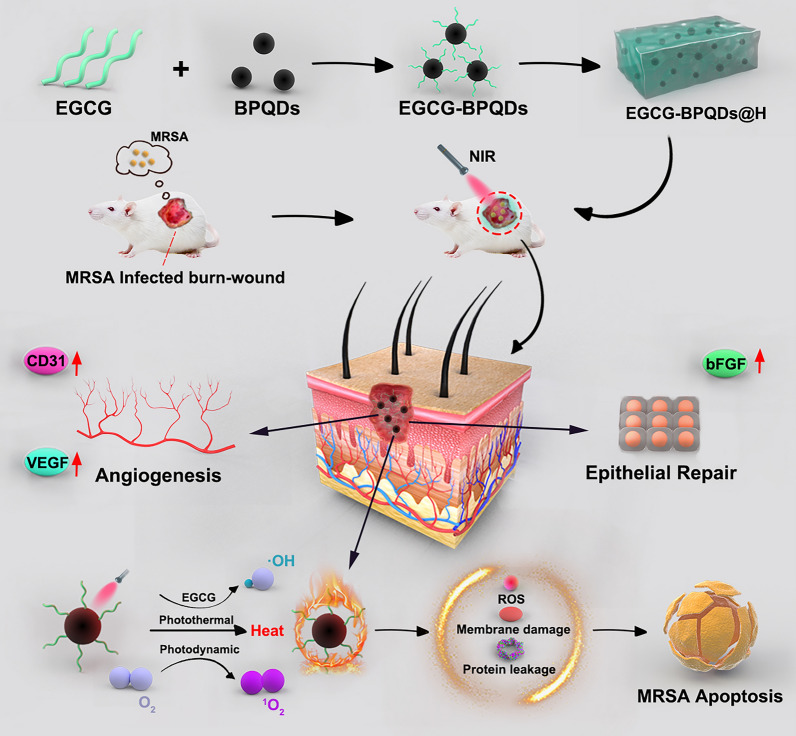

**Supplementary Information:**

The online version contains supplementary material available at 10.1186/s12951-021-01106-w.

## Introduction

MRSA infected deep-burn wound in diabetic patients is a fatal threat to human health and the frontier in clinical medicine [1]. Surgical debridement and continuous use of antibiotics are still the primary clinical treatment for MIDBW [2, 3]. However, suboptimal pharmacokinetics and large doses of antibiotics usually cause severe side effects, like deadly complications of resistant bacteria infection [4]. MRSA, which causes wound infections, is currently a major obstacle to the healing of diabetic wounds. It can even cause amputation of the patient's limb [5, 6]. Even worse, the development of new antibiotics has not been realized for a long time worldwide [7]. Therefore, besides surgical debridement and continuous use of antibiotics, multifunctional collaborative therapy nanoplatform attracts MIDBW in diabetic patients. To date, various nanomaterials have been currently available in diabetic-infected wound healing, such as metal–organic frameworks [8, 9], dopamine [10–13], peptides [14], metal oxides, and nano silver [15, 16]. These excellent pioneered works suggest that multifunctional nanoplatforms with advantages of strong antibacterial and effective promoting of wound healing function are attractive, promising strategies for treating infected wounds.

Among these numerous antibacterial materials, black phosphorus (BP) is attractive because of its broad optical absorption and striking charge carrier mobility, exhibiting outstanding singlet oxygen (^1^O_2_) activation and the high-temperature generation under NIR irradiation [17, 18]. Recently, photocatalytic properties and photothermal activity have been intensively investigated in biomedical applications [19], especially in the field of anti-infection wound treatments by their virtues of minimally invasive, effective sterilization, and easy operation [20–22]. These reported excellent works disclosed the attractive prospect of BP in infected wound healing. However, these documents mostly focused on BP nanosheets, while BPQDs have seldomly been researched, particularly in MIDBW. Therefore, the construction of a multifunctional BPQDs nanoplatform with collaborative properties of NIR response, ROS generation, sterilization, and promoted wound healing is a challengeable but attractive strategy for MIDBW therapy.

**Scheme 1.**
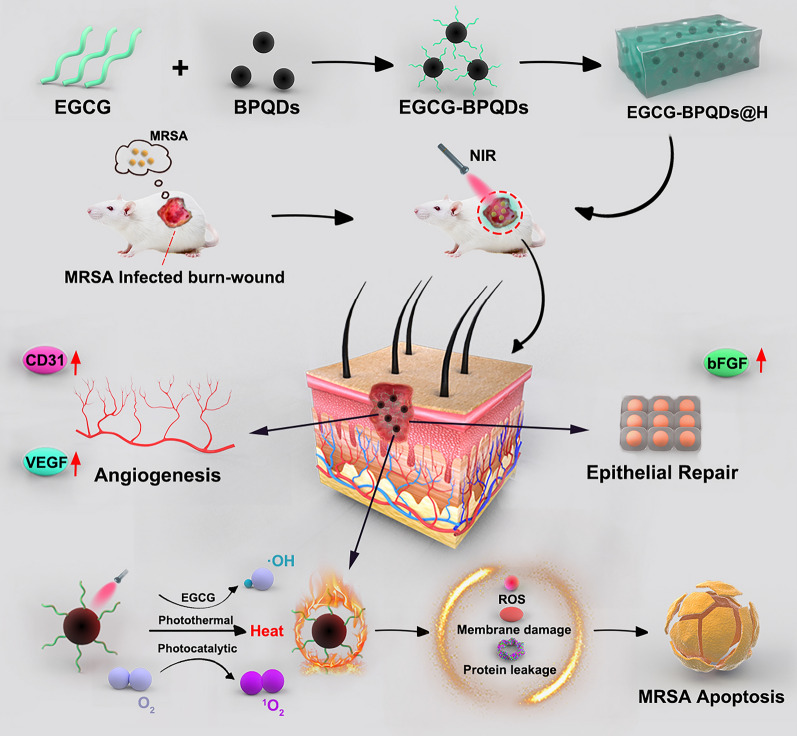
The schematic illustration of the synthesis of EGCG-BPQDs@H nanocomposite and the process of sterilization and stimulation of cell behaviors that can promote regenerative activities of the skin cells and actively participate in epidermal regeneration, and accelerate the healing in diabetic infected wounds

Herein, in order to achieve a multifunctional BPQDs nanoplatform, we proposed a strategy of constructing EGCG-modified BPQDs. As illustrated in Scheme 1, firstly, EGCG molecules were modified on BPQDs by the interactions between -OH group on EGCG and P atoms from BPQDs, such as hydrogen bond and molecule interactions. Previous works have proved the excellent biological function of tea polyphenol, especially EGCG [23–26]. Then, the obtained EGCG-BPQDs were loaded into hydrogels as a dual-purpose nano-agent to possess an antibacterial effect and simultaneously accelerate wound healing. Specifically, the engineered nanomaterials are expected to promote angiogenesis and epithelial regeneration by upregulating cellular CD31 and bFGF expression. Furthermore, under NIR irradiation, the nanomaterials will induce high levels of ROS production to eradicate antibiotic-resistant bacterial infections while generating local heat and accelerating microcirculatory blood flow. Our previous works and related reported works have explored hydrogels that can be used as excellent drug delivery systems [27]. Hydrogels encapsulated with EGCG-BPQDs will provide sustainable moisture and prolonged release of EGCG-BPQDs, which are beneficial for wound healing. Finally, the constructed hydrogels encapsulated EGCG-BPQDs are expected to achieve functional antibacterial, vascular regeneration, and accelerated epithelial regeneration.

## Materials and methods

### Materials

Methicillin-resistant Staphylococcus aureus (MRSA) and Gram-negative bacteria Escherichia coli (*E. coli*) were purchased from Guangdong Microbial Culture Collection (China). Dulbecco's modified Eagle's medium (DMEM), 3-(4,5-Dimethylthiazol-2-yl)-2,5-diphenyl tetrazolium bromide (MTT), and fetal bovine serum (FBS) were purchased from Gibco (USA). Human skin keratinocytes cells (HaCat) and human umbilical vein endothelial cells (HUVEC) were obtained from American type culture collection (ATCC). The 2,2’-bis(anthracene-9,10-diylbis(methylene))-dimalonic acid (ABDA) was supplied from Shanghai Civi Chemical Technology Co., Ltd. 1-methyl-2-pyrrolidone (NMP) was purchased from Aladdin. The primary antibodies to CD31, VEGF, bFGF, Tubulin were obtained from Cell Signaling Technology (USA). We used deionized water throughout the experiment.

### Characterization

The component of EGCG-BPQD@H was identified by Fourier transform infrared spectroscopy (FTIR, SHIMADZU, Kyoto, Japan) with the KBr disk method. Relevant fluorescence data were acquired with a fluorescence photometer (F97PRO, Shanghai, China). UV–vis data were measured by UV–vis spectrophotometer (PerkinElmer Lambda 605S UV–vis spectrometer) at room temperature. The sample's crystal structure was tested by X-ray diffraction (XRD, Shimadzu, Kyoto, Japan) and Cu K radiation. We also used dynamic laser scattering (DLS, Malvern, Nano ZS90, Worcestershire, UK) to test the sample's particle size. The sample's appearance characteristics were displayed through a transmission electron microscope (TEM, JEM-1200EX, Tokyo, Japan). Infrared thermal imager and NIR laser were purchased from Dongguan Bufan Electronics Co., Ltd., China. Confocal laser scanning microscopy (CLSM, Leica TSCSP5 confocal unit) was used to observe the expression of related proteins in cells.

### Therapy of burn wounds infected with MRSA in diabetic rats

All animal experiments were carried out according to the Guidelines for Care and Use of Laboratory Animals of Jinzhou Medical University. Male Sprague–Dawley rats, weighing 190 ± 20 g, were obtained from Vital River Company (Beijing, China). After one week of acclimatization at the animal holding unit, the rats were ready for the following experiments. The diabetic animal model was established with streptozotocin (STZ; St. Louis Sigma, Missouri; 80 mg/kg), and rats were injected intraperitoneally with STZ (0.1 M pH 4.5 citrate buffer formulation) once daily for four days. Rats with blood glucose levels greater than 300 mg/dL were selected for the test. Briefly, the dorsal hair was shaved and decontaminated after anesthetization, and then the electric device heated at 120℃ was in contact with the shaved dorsal skin for 12 s to cause a diameter of ~ 13 mm deep partial-thickness burn injury. Then the burn area was injected 100μL of MRSA suspension and was diluted to 2.0 × 10^7^ CFU/mL to build an experimental model of infection. After continuous infection for three days, the prepared hydrogel, BPQDs@H, EGCG@H, and EGCG-BPQDS@H were applied to the wound once daily directly. Meanwhile, the infected wound area was photographed at the same height, and a vernier caliper measured the size of wounds. The control + NIR, BPQDs@H + NIR, EGCG@H + NIR, and EGCG-BPQDS@H + NIR groups were treated with 808 nm laser (2.5 W/cm^2^, 5 min), and the temperate changes of wounds were monitored via thermographic pictures captured by the infrared thermal imaging system. Then bodyweight of rats was registered every time. The burn wounds tissues and systemic organs were harvested for protein extraction and histological analyses.

### In vitro antibacterial activity analyses

After activation and incubation, MRSA and *E. coli* were cultured at 37℃ in a fresh liquid LB medium. The MRSA and *E. coli* (1.0 × 10^8^ CFU/mL) were briefly extended into the LB-Agar-Medium. After that, filter papers containing EGCG-BPQDs@H or other nanomaterials were spread on the MRSA and *E. coli* agar plates as a method of antibacterial activity evaluation. Each group’s diameters of the bacteriostatic ring were measured after incubation for 24 h. We also used MRSA and *E. coli* to estimate the synergetic antibacterial effects of EGCG-BPQDs@H plus NIR laser irradiation. Logarithmic growth phase bacteria were incubated in LB medium and then mixed with each group of materials in a 1.5 mL Solarbio tube containing 100μL of normal saline. For all groups, the bacteria were illuminated with/without NIR laser (808 nm, 2.5 W/cm^2^) for 10 min and were incubated for another 24 h in the LB-Agar-Medium for CFU. In addition, to observe the antibacterial effect of different groups directly, bacterial morphology was examined by scanning electron microscope (SEM).

### Calcein-AM/PI staining

The antibacterial effect was measured by the double fluorescent dye method. Specifically, bacteria were treated with hydrogel, BPQDs@H, EGCG@H, or EGCG-BPQDs@H for 3 h at 37℃. The bacteria were irradiated with/without NIR laser (2.5 W/cm^2^, 10 min). Bacteria were incubated with calcein-AM and PI (Solarbio, China) after centrifuging at 5000 rpm for 5 min. Then, the bacteria were washed with phosphate-buffered saline (PBS) and placed on glass slides. The bacteria with different treatments were presented under a fluorescence microscope (Leica DM4000B, Germany). The red-fluorescent nucleic acid dye PI, which can penetrate the damaged cell wall, was used to mark dead bacteria. In contrast, the Calcein-AM that penetrated the living cell membrane was cleaved by the intracellular esterase to form Calcein, which emitted strong green fluorescence and remained in the live bacteria.

### Photocatalytic property of EGCG-BPQDs@H

The ABDA probes were applied to detect singlet oxygen generation and time-varying changes. The fluctuation of ABDA probes was recorded to reflect the generation of ROS. We added EGCG-BPQDs@H into the 5 mM ABDA solution and then irradiated it under the near-infrared laser. The test was performed every 5 min. In addition, the single linear state of oxygen produced is detected by electron spin resonance spectroscopy.

The intracellular ROS level of bacteria was measured by fluorescent probe, 2′,7′-dichlorodihydrofluorescein diacetate (DCFH-DA, Beyotime, China), which could be deacetylated and oxidized to fluorescent products. The MRSA, after various treatments, was fixed with 4% paraformaldehyde and then incubated with DCFH-DA probe for 30 min in the dark. The fluorescence imaging was immediately recorded with Leica DM4000B microscope. The fluorescent intensity was measured via ImageJ and Fiji12 plugin to quantify the total ROS level via EGCG-BPQDs@H.

### Determination of protein leakage

The leakage of protein through the membrane of MRSA was observed after exposing different samples. NIR-treated groups were irradiated with 808 nm NIR light for 20 min at a power density of 2.5 W/cm^2^. Subsequently, the bacterial suspension supernatant was collected by centrifugation at 12,000 rm for 5 min. Finally, the supernatant liquid was immediately withdrawn and seeded into a 96-well plate. The protein intensities of different groups were determined by the microplate reader at 562 nm through the BCA assay kit (Beyotime, China) to determine the relative protein leakage of each sample.

### Biofilm formation assay

MRSA biofilm model was employed to assess the antibiofilm ability of EGCG-BPQDs@H antibacterial nanoplatforms. 2 mL of 5 × 10^8^ CFU mL^−1^ of MRSA were seeded into a 24-well plate and incubated for 48 h to establish biofilm. After different treatments, crystal violet (CV) staining was utilized to evaluate the antibiofilm effect. MRSA biofilms were washed with gentle running deionized water to remove the unbound dye. The bound CV was dissolved using absolute ethanol. At last, a microplate reader was used to measure the absorbance of all samples at 590 nm.

### Histology analyses

In the infected burn wound healing experiment, individual wound tissues and major organs of the rats were harvested for histological analysis. The tissues were fixed in 4% formaldehyde and dehydrated through alcohol. The paraffin-embedded tissues were cut to a 5 μm thickness and were stained with hematoxylin and eosin (H&E) and Masson stain to evaluate the recovery of wound healing. Besides, the biocompatibility of nanomaterials in vivo as assessed by H&E staining of main organs. The sections were captured with Leica DM4000B microscope.

### In vitro angiogenesis assay of HUVECs

The angiogenesis ability of EGCG-BPQDs@H in vitro was assessed by endothelial tube-like formation as specified by the manufacturer’s guidelines. HUVEC was used for this assay. The night before the experiment, pipette tips and well plates were placed in a − 20 ℃ freezer, and the Matrigel (BD Biosciences, USA) thawed at 4℃. First, 80 μL Matrigel was incubated into a 96-well plate for 30 min at 37 ℃ to induce gelation. 1.0 × 10^4^ HUVECs were first seeded on EGCG-BPQDS@H or BPQDs@H or EGCG@H with 100μL of FBS-free culture medium above solidified Matrigel for 6 h. finally, the differentiation of HUVECs were observed through an inverted phase-contrast microscope (Leica DM4000B, Germany). The total length and number of nodes and segments were digitally imaged and quantified using the Angiogenesis Analyzer macro in ImageJ.

### In vitro HUVECs migration experiment

Scratch test to assess the migration ability of HUVECs, which measures the expansion of HUVECs on margin. HUVECs were seeded at a density of 8 × 10^4^/well in a 12-well plate with FBS-free medium to form a confluent monolayer. After incubation for 1 d, a straight scratch was created by pipette tip, and the debris was gently washed twice with PBS. Then, each group was treated with their corresponding nanomaterials at 37 ℃ in the incubator. The cells were treated with 4% paraformaldehyde, incubated with 0.1% Triton X-100 (Sigma, USA), and then stained with 4′,6-diamidino-2-phenylindole solution (DAPI, Invitrogen, USA). The difference between scratch wounds at 0 h and 24 h was captured with an inverted microscope, and the rate of cell migration was calculated as follows:

$${\text{HUVECs migration }}\left( \% \right) \, = \, \left( {{\text{W}}_{{0{\text{h}}}} - {\text{W}}_{{{\text{12h}}}} } \right)/{\text{W}}_{{0{\text{h}}}} ,1$$

W_0h_ was the initial wound area, and W_12h_ was the wound area after 12 h of incubation.

### Cell viability assay

The MTT assay was used to determine the proliferation of HUVECs in leach liquor of each group material and normal medium at different times. Firstly, HUVECs (5000 cells/well) were incubated in 96-well plates for 24 h. After starvation with fetal bovine serum (FBS)-free medium overnight, the culture medium was removed and replaced by the different groups’ leach liquor, respectively. They were incubated, and the solution in each well was pipetted out at 6, 12, and 24 h. Subsequently, 20μL of MTT solution (5 mg/mL in FBS) was added to each well for 4 h at each time. Then, the supernatant was removed, and 150μL dimethyl sulfoxide (DMSO) was added to each well and incubated for 15 min in a dark place. Finally, quantitative detection was performed on a microplate reader at 490 nm. In order to better mimic the microenvironment of bacterial infections in vivo, the cells were pretreated with 1 μg/mL lipopolysaccharide (LPS) followed by different nanomaterials treatments for 24 h, and cells treated with LPS alone were used as a positive control.

### Western blot analysis

Cells or tissues around the wound were collected and homogenized with RIPA (EnoGene, China) buffer to collect supernatants. Equal amounts of proteins (15 μg) separated on 10% polyacrylamide gels and transferred on PVDF membrane, which was blocked with 5% skim milk for 2 h. Then, the membranes were incubated with CD31, VEGF, bFGF, Tubulin antibodies followed by corresponding second antibodies for 2 h at indoor temperature. Immune reactivity was detected with a super signal ultra-chemiluminescent reagent (Pierce Chemical, Rockford, IL, USA). Images were captured by Alpha Innotech Photo Documentation System (Alpha Innotech, Hayward, CA, USA). Quantification of the protein expression was performed by ImageJ software.

### Immunofluorescence double labeling method

The leach liquor of each group was added to the well plate. In each group, HUVECs and HaCaTs were washed 3 times with PBS and treated with 4% PFA for 40 min. After incubated with 0.1% Triton X-100, cells were washed 3 times and blocked with 5% goat serum for 2 h. Cells were also incubated with primary anti-bFGF (1:1000, CST, USA); anti-Tubulin (1;1000, CST, USA); anti-CD31 (1;500, Abcam, UK); anti-VEGF (1;500, Abcam, UK) overnight at 4℃. Subsequently, they were washed 3 times and incubated with Alexa Fluor 488 goat anti-rabbit IgG or Alexa Fluor 594 goat anti-mouse IgG (1;500, Thermo, USA) for 2 h. Nuclei were stained with DAPI for 15 min. After washing, confocal laser scanning microscopy was used for other cells characterization.

### Statistical analysis

All independent experiments were performed in triplicate in the corresponding condition. All Graphs were rendered using GraphPad Prism software, version 6. One-way analysis of variance (ANOVA) with post hoc Tukey multiple comparison tests was used to analyze statistical significance. Differences between different groups at *P < 0.05, **P < 0.01, ***P < 0.001 were considered as statistically significant.

## Results and discussion

### Preparation and characterization of EGCG-BPQDs@H

**Fig. 1:**
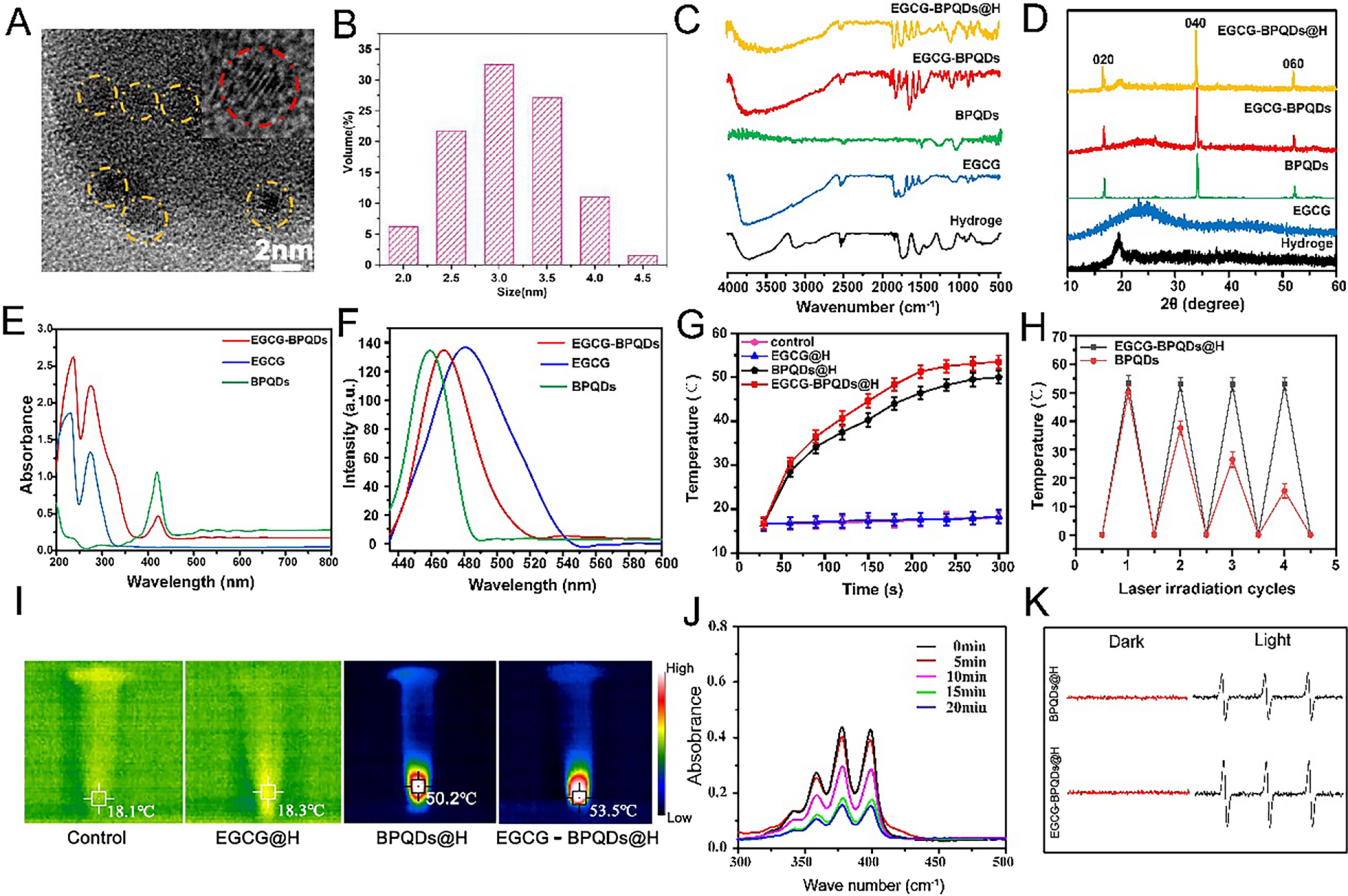
Characterization of BPQDs@H, EGCG@H, EGCG-BPQDs@H. **A** HRTEM images and **B** DLS size distribution of EGCG-BPQDs; the insert in A is the image of one single magnified BPQD. **C** FTIR spectra, **D** XRD, **E** UV–vis and **F** fluorescence emission (λex = 400 nm) of samples. **G** The temperature rises curves of four groups of samples within 5 min. **H** The thermal curves of BPQDs@H and EGCG-BPQDs@H after repeated laser irradiation (n = 3). **I** Infrared thermography images of samples under 808 nm laser irradiation for 5 min. **J** ABDA solution containing EGCG-BPQDs@H after irradiation for different times. **K** The ESR spectra of BPQDs@H and EGCG-BPQDs@H after being in the dark or irradiated with light for 10 min

The black phosphorus quantum dots were obtained by the liquid-phase ultrasonic stripping method, and 500 mg of black phosphorus powder was put into 50 mL of N-methyl pyrrolidone (NMP), mixed, and ground in a mortar. The mixture was sonicated in an ice bath for 4 h, and then centrifuged at a speed of 12,000 r/min for 20 min. The supernatant containing BPQDs was decanted. We then carried out a series of related experiments to verify the successful preparation of the composite. Firstly, we used HRTEM to observe the morphological characteristics of EGCG-BPQDs. As shown in Fig. 1A, we can see homogeneous EGCG-BPQD nanoparticles. The insert in Fig. 1A is the image of one single magnified BPQD. The lattice fringes of the BPQD could be discriminated. The DLS indicated that the size of BPQDs is about 3 nm (Fig. 1B).

The results of FTIR spectra showed that the absorption peak at 1718 cm^−1^ and the broad absorption peak at 1400–1600 cm^−1^ were characteristic absorption peaks of -C=O and benzene of EGCG, 1100 cm^−1^, and 1250 cm^−1^ were P=O stretching vibration peak. It is proved that the prepared EGCG-BPQD@H already contains EGCG and BPQD (Fig. 1C). In addition, the synthetic hydrogels were tested by FTIR (Additional file 1: Fig. S1). We tested the crystal form of the synthesized product by XRD. In Fig. 1D, the diffraction peaks at ~ 16°, 34°, and 52° corresponded well to the diffraction peaks of the (020), (040), and (060) crystal planes of BPQD, showing that BPQD still maintains a complete structure when it was fused to the hydrogel or bonded to EGCG (Fig. 1D). We demonstrated the successful preparation of EGCG-BPQDs by UV–vis spectroscopy. As shown in Fig. 1E. BPQDs exhibited a strong and sharp absorption peak at approximately 420 nm, which indicated the successful synthesis of high-quality BPQDs [28]. Moreover, the characteristic absorption peaks of EGCG and BPQDs can be observed in EGCG-BPQDs, which again demonstrated that we have successfully synthesized EGCG-BPQDs. The fluorescence spectra demonstrated that the emission peaks of BPQDs and EGCG appear at 460 nm and 481 nm, respectively, and the peak of EGCG-BPQDs@H appears at 467 nm (Fig. 1F). These results indicated that we have successfully prepared EGCG-BPQDs and well dispersed in hydrogels.

To verify the photothermal capability of EGCG-BPQDs@H in photothermal therapy, we irradiated EGCG-BPQDs@H with a NIR laser (2.5 W/cm^2^, 808 nm) for 5 min and recorded the temperature change with a thermal imager (Fig. 1G and I). As shown in Fig. 1H, the temperature of EGCG-BPQDs@H and BPQDs@H increased to 53.5 ℃ and 50.2 ℃, while the temperature of EGCG@H only changed to 1.7 ℃, indicating that EGCG-BPQDs@H have excellent photothermal performance while its maximum photothermal conversion efficiency was reaching 46.7% (Additional file 1: Fig, S2). Finally, the stability of EGCG-BPQDs@H under cyclic light was also further explored. The samples were irradiated for the same duration and cycle (4 cycles, 5 min each cycle). The highest temperature that the samples could reach after each cycle was recorded using a thermal imager (Fig. 1H). At the second cycle, the maximum temperature of the BPQDs began to decrease rapidly, reaching a maximum temperature of just 15.5 ℃ by the fourth cycle. In contrast, maximum temperature of the EGCG-BPQDs@H remained near 52 ℃ and still maintained good photothermal properties. These results indicate that the hydrogels provided some protection to the BPQDs and reduced the degradation of the BPQDs.

The production of singlet oxygen is an important basis for assessing the effectiveness of the photocatalytic effect with photosensitizers. We evaluated the ability of EGCG-BPQDs@H to produce singlet oxygen under NIR irradiation using ADBA solution. Under NIR conditions, the intensity of the absorption peak of the ABDA solution at 380 nm decreased sharply, indicating that the material produces a large amount of singlet oxygen (Fig. 1J). In contrast, the absorption spectra of the ABDA solution alone or the EGCG@H group did not change significantly under NIR irradiation (Additional file 1: Fig. S3A, B). To further validate the conclusions, electron spin resonance measurements were performed to verify the ability of the material to produce singly linear oxygen. After 5 min of irradiation, typical electron spin resonance spectra were observed (Fig. 1K). The relative intensity of the electron spin resonance produced by EGCG-BPQDs@H was significantly higher than BPQDs@H for the same recording time, indicating that EGCG-BPQDs@H had a stronger ability to produce singlet oxygen. In vitro release experiments with EGCG@H were performed using dialysis bags and demonstrated that free EGCG showed a faster release rate. In contrast, EGCG@H and EGCG-BPQDs@H could control the slow and smooth release of EGCG (Additional file 1: Fig. S3C). UV–vis indicated that more EGCG was released from EGCG-BPQDs@H under NIR irradiation, which contributed to our further modulation of drug release (Additional file 1: Fig. S3D).

### In vitro antibacterial properties of EGCG-BPQDs@H

**Fig. 2:**
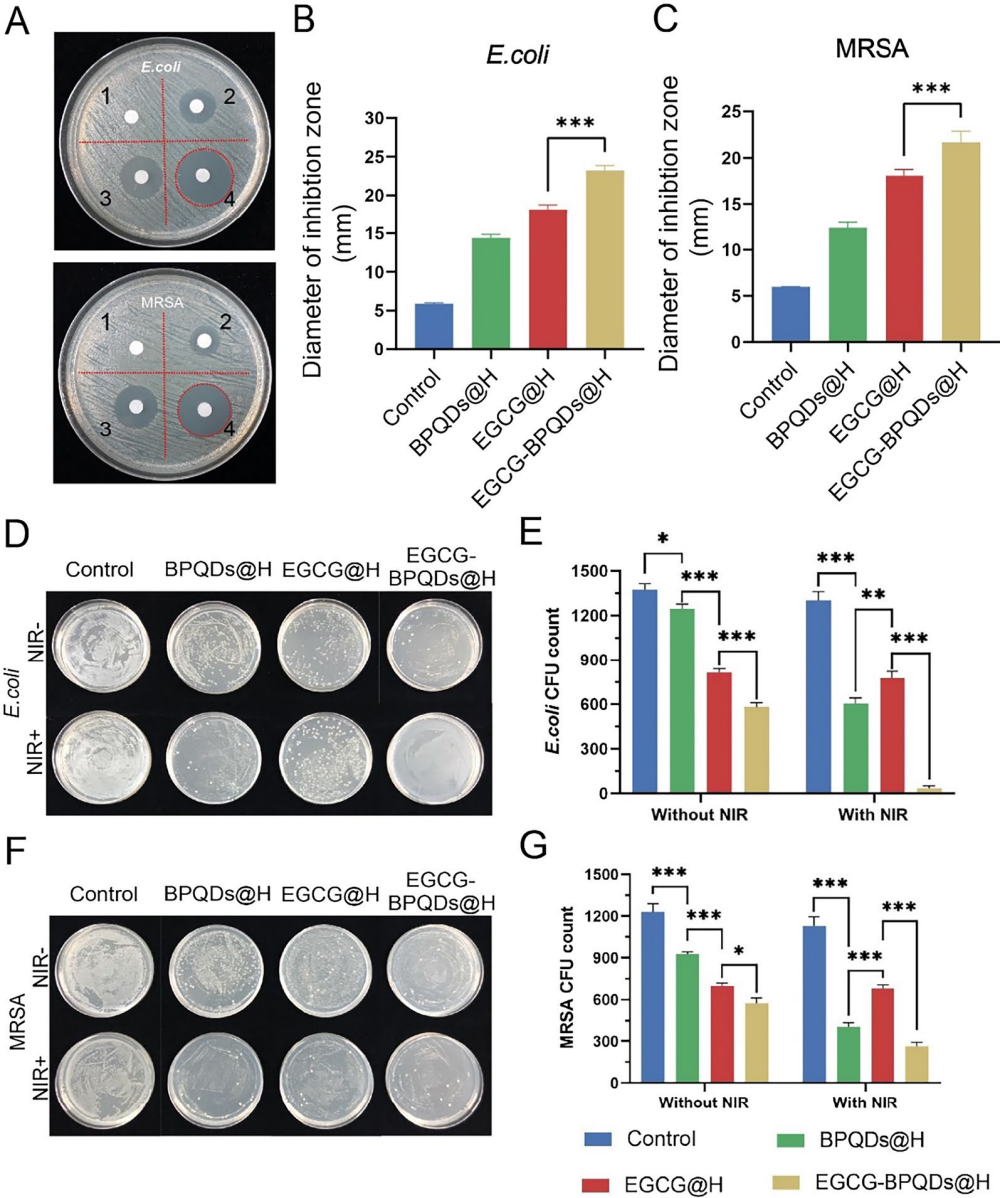
Antimicrobial efficacy of various methods on MRSA and *E. coli*. **A** Inhibition zones and **B**, **C** the corresponding statistical graphs of MRSA and E. coli with different treatments (1: hydrogel, 2: BPQDs@H, 3: EGCG@H, 4: EGCG-BPQDs@H). **D**, **F** Digital photos of bacterial colonies on agar plates with different treatments. **E**, **G** The corresponding bacterial colonies on agar plates were calculated

The agar spread assay was performed to evaluate the antimicrobial capacity of different nanomaterials. After 37℃ constant temperature cultivation, treatment with hydrogel did not show antibacterial effect for the tested bacterial. On the contrary, antibacterial rings of tested bacteria were clearly observed after treatment with BPQDs@H, EGCG@H, or EGCG-BPQDs@H (Fig. 2A). The diameters of the EGCG-BPQDs@H group antibacterial zone towards *E. coli* and MRSA were ~ 23 mm, ~ 21 mm, respectively (Fig. 2B and C), demonstrating its obvious antibacterial activity. These results presented a stronger antibacterial effect after treatment with EGCG-BPQDs@H, attributed to the synergistic relationship between EGCG and BPQDs. In addition, experiments demonstrated that BPQDs could cause bacterial toxicity in the absence of NIR. MRSA and *E. coli* cells from different treatments were diluted and spread on the agar plates, respectively. Next, the colony counts assay investigated the antibacterial effect of black phosphorous quantum dots (Fig. 2D and F). The results of the colony count assay without NIR were in harmony with the antibacterial ring. Nevertheless, the bacteria viability in BPQDs@H + NIR treated groups was only 3.2% for E. coli and 11.3% for MRSA, respectively (Fig. 2E and G).

These data also suggested the stronger antibacterial effect empowered by phototherapy. Anyhow, these results indicated that neither EGCG-BPQDs@H nor photothermal treatment could eliminate the selected bacteria. On the contrary, especially in E. coli, almost no bacterial colonies were detected after combinational treatment EGCG-BPQDs@H under NIR irradiation. That is to say, the EGCG-BPQDs@H + NIR could more efficiently suppress bacterial viability and inhibit the growth of E. coli and MRSA. Briefly, the local thermal heat produced by nanomaterials under NIR irradiation was able to destroy bacteria by deactivating enzymes and disrupting metabolism in bacteria. Moreover, the NIR radiation provoked the release of EGCG from EGCG-BPQDs@H, which increased the local EGCG concentration. The high EGCG content affected bacterial activity and led to bacterial death [29]. Therefore, cooperative treatment was a prominent strategy to eliminate bacteria.

### Evaluation of the integrity of bacteria

**Fig. 3:**
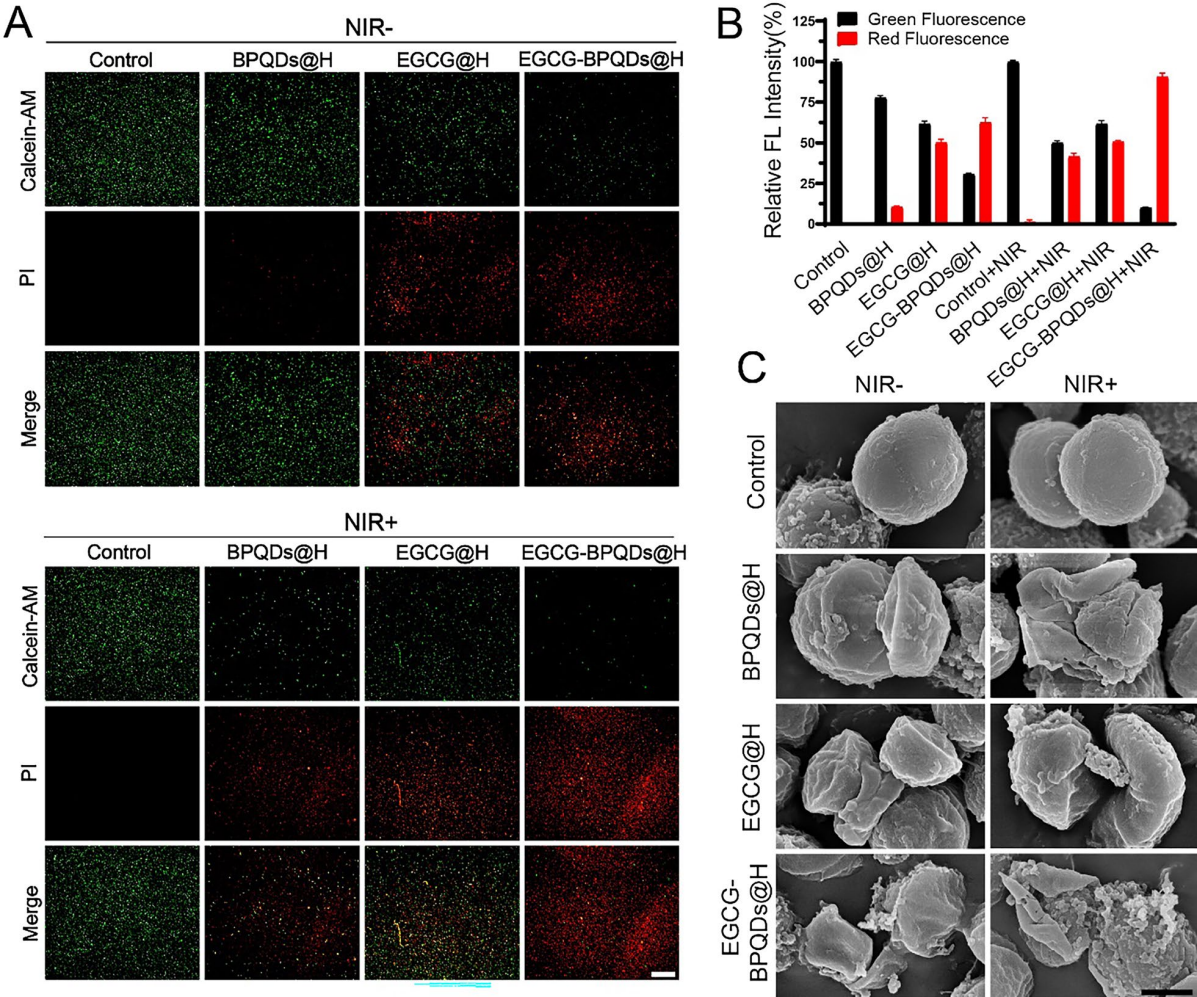
Deformation of the external structure of bacteria disposed of by EGCG-BPQDs@H. **A** Calcein-AM/PI staining images of MRSA in different treatment groups. Red spots signify dead bacteria, bar = 50 μm. **B** The corresponding quantitative assay of live and dead bacteria. **C** The SEM images, bar = 500 μm

The bacterial survival rate was further identified through fluorescence staining assays to evaluate the antibacterial effect of the cooperative treatment. Fluorescence pictures of live/dead staining showed that only intense green fluorescence and no visible red fluorescence were observed in bacteria after treatment with normal hydrogels or with NIR only. By contrast, the BPQDs@H, EGCG@H, EGCG-BPQDs@H, BPQD@H + NIR, and EGCG@H + NIR treated bacterial emitted red fluorescence as well as green fluorescence, suggesting regional antibacterial efficacy was achieved (Fig. 3A). The strongest red fluorescence could be detected in the group in which the bacterial were treated with EGCG-BPQDs@H with NIR, indicating the potent antibacterial activity of the cooperative treatment (Fig. 3B). SEM was used to observe the cellular morphological changes of MRSA following the different treatments (Fig. 3C). As demonstrated in SEM pictures, it was found that bacterial remained.

typical spherical shape and intact smooth surface after treatment with normal hydrogel, NIR. In the case of the MRSA treated with BPQDs@H, EGCG@H, EGCG-BPQDs@H, BPQD@H + NIR, or EGCG@H + NIR, obvious disruption appeared on their original morphology, indicating their surface became slightly rough and fragmentary. In contrast, the structure of bacteria was distorted and displayed more serious damage after combining with EGCG-BPQDs@H under NIR irradiation, implying the strongest antibacterial effect of the cooperative treatment. Besides the special photothermal property of BPQDs can convert near-infrared light into local high temperature to denature the enzymes in the bacteria, the phenolic hydroxyl of EGCG could bind to the phospholipid bilayer of bacteria and the amino and carboxyl groups in bacterial membrane proteins, which was reported to disrupt the integrity of the bacterial membrane [30].

### Antibacterial mechanism exploration in vitro

**Fig. 4:**
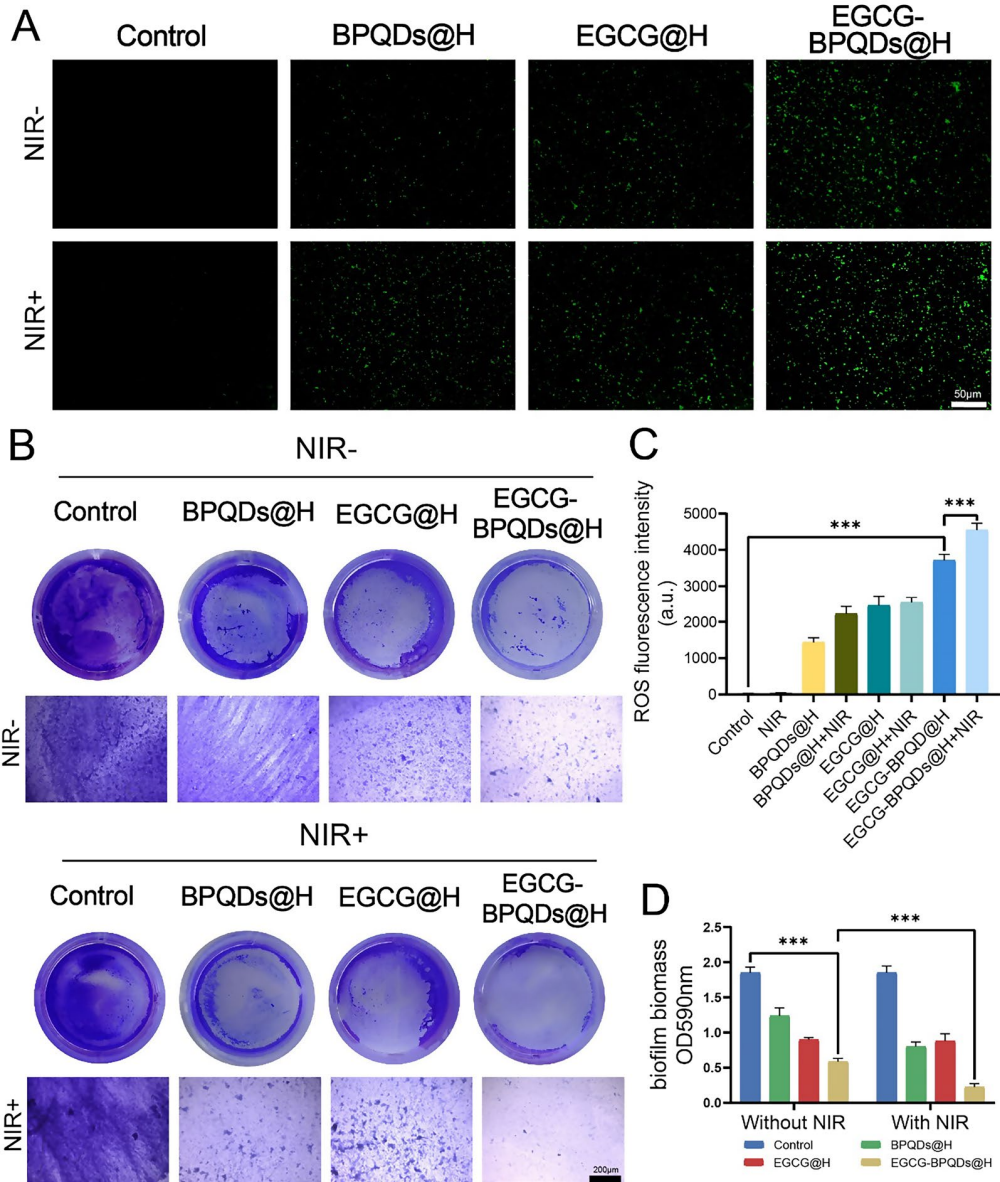
The investigation of antibacterial mechanisms. **A** Fluorescent photographs of ROS level in MRSA with various treatments in vitro, stained by DCFH-DA, bar = 100 μm. **B** Crystalline violet-stained biofilm. **E** Images of MRSA biofilms stained with crystal violet, bar = 200 μm. **C** Consequential statistics of histograms indicating religious ROS activity levels. **D** Absorbance of biofilm in different groups

In addition to the above results, the active oxygen generated under radiation was further examined to explore the antibacterial mechanism and antibacterial effect. DCFH-DA was used to monitor the generation of ROS after different treatments on MRSA. As a potential photocatalyst, black phosphorous nanomaterials could generate numerous singlet oxygen (^1^O_2_), introducing reactive oxygen-dependent oxidative stress and membrane damage [31–34]. It was observed that little green fluorescence of the bacteria was detected after treatment with normal saline or NIR. Whereas the laser irradiation significantly enhanced ROS production in BPQDs@H and EGCG-BPQDs@H treated group. More importantly, a much higher level of ROS was generated in EGCG-BPQDs@H treated group, partly attributed to the spontaneous slow release of EGCG. Meanwhile, comparing with EGCG-BPQDs@H group, ROS production was obviously upregulated in EGCG-BPQDs@H + NIR group due to the EGCG release and high temperature (Fig. 4A and C). Since the rupture of the membrane causes cytoplasm to leak, the degree of protein leakage can be used as a response to the antibacterial effect [35]. We further evaluated the leakage of intracellular protein after exposure to different groups. As shown in Additional file 1: Fig. S4, the leakage of proteins from the bacteria treated with the combination was significantly reduced. Nevertheless, in the other groups, only relatively weak changes were observed, indicating that EGCG-BPQDs@H caused severe disruption of the bacterial cell membrane under NIR irradiation.

Biofilms have further weakened the therapeutic effect due to their resistance to antibiotic therapy [36]. Therefore, the antibacterial activity of EGCG-BPQDs@H against the MRSA biofilm was the further test. The darker the blue, the more biofilm stained by crystal violet. Limited radicalization was observable following normal saline or BPQDs@H treatment alone, and the radicalization activity was not related to NIR light (Fig. 4B). As shown in Fig. 4D, the corresponding absorbance and solution color of the group treated with EGCG-BPQDs@H + NIR were the least of all the treating sets, suggesting that the synergistic system of EGCG-BPQDs@H + NIR has an efficiency against biofilm. In brief, simply NIR radiation, EGCG@H, or the BPQDs@H cannot kill bacteria effectively. Under the NIR irradiation, the photothermal and photocatalytic properties of EGCG-BPQDs@H could be activated, together with increased release of EGCG, which exerted additional antibacterial effects to the bacteria system. EGCG-BPQDs@H excited by NIR laser present a higher bactericidal efficiency than EGCG-BPQDs@H. This enhancement might be due to the synergistic effect of photothermal and active oxygen sterilization. Comparison between EGCG-BPQDs@H and BPQDs@H also affirmed the positive efficacy of EGCG, and there was no significant change before and after NIR illumination. Accordingly, the NIR regulated photothermal, ROS and release promoting synergetic therapy could be used to completely combat multidrug-resistant bacterial infections. In addition, we obtained fluorescent images of bacterial biofilms after incubating the composite hydrogel-treated bacteria with FITC-Con A (Additional file 1: Fig. S5). The bacterial biofilm was significantly reduced after treatment with EGCG-BPQDs@H with NIR irradiation, demonstrating the good antibacterial properties of this hydrogel system for biofilms.

### Promoting migration and proliferation effects of EGCG-BPQDs@H in vitro

**Fig. 5:**
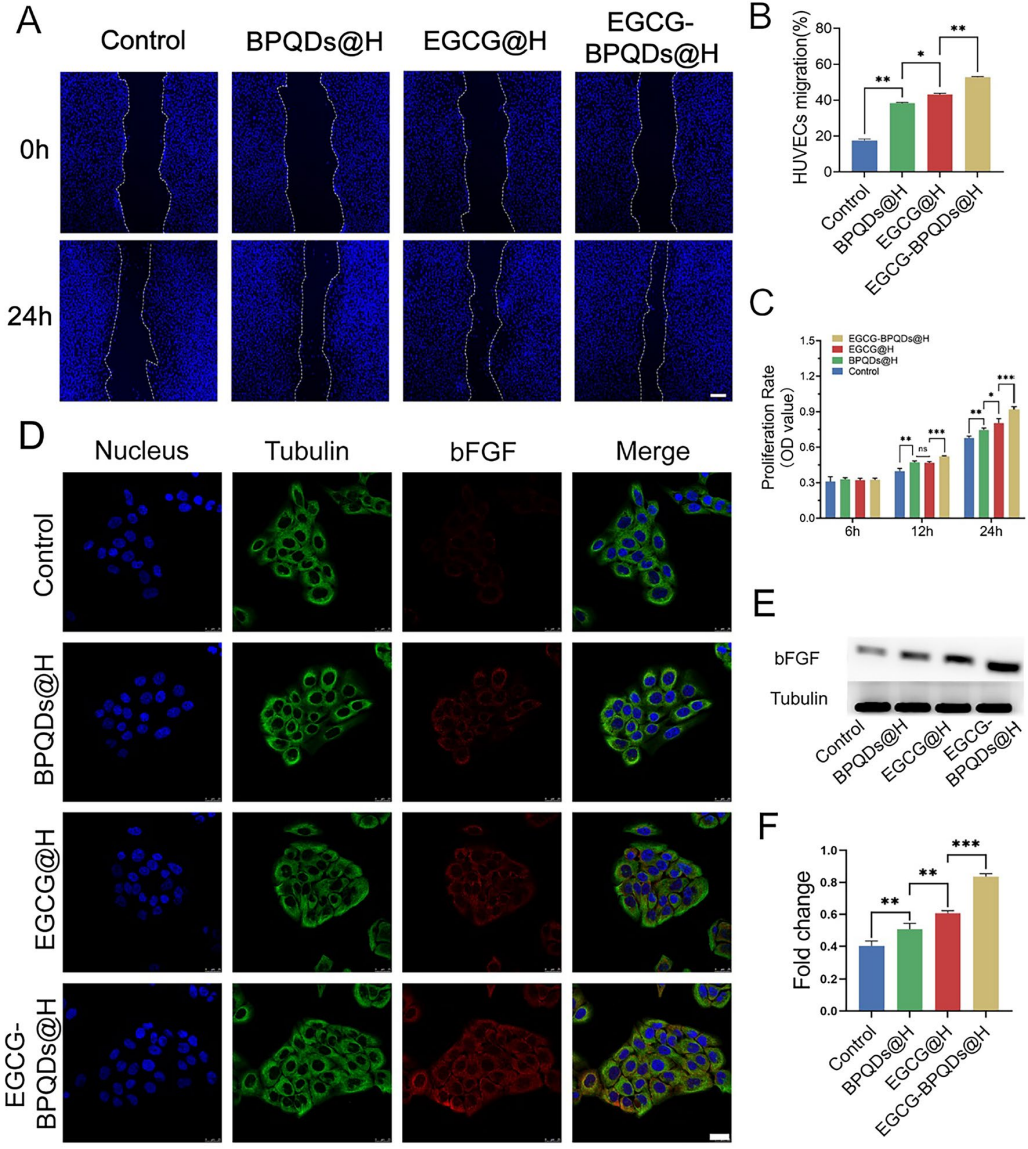
Promoting migration and proliferation effects and potential mechanism. **A** Digital images of scratch wound assay in different treatments, bar = 100 μm. **B** Quantification of HUVEC migration. **C** MTT analysis in different groups. **D** CLSM images of the expression analysis of bFGF, bar = 25 μm. **E** The expression level of bFGF protein. **F** Quantification analysis of the bFGF protein. *P < 0.05, **P < 0.01, ***P < 0.001

It is commonly accepted that endothelial cell migration is critical in angiogenesis [37]. EGCG is considered to have a superior impact in accelerating wound healing by virtue of antibacterial, anti-inflammatory, and facilitating angiogenesis [38–40]. Therefore, the regenerative ability of EGCG-BPQDs@H was further investigated through HUVECs. Following the different treatments of cells scratch, the migration extent revealed a difference (Fig. 5A). Compared with other groups, significant cell migration was detected in EGCG-BPQDs@H group after 24 h incubation (Fig. 5B). The wound area in EGCG-BPQDs@H group decreased dramatically by 52.8%, while only 43.1%, 38.3%, and 17.6% of the wound decreased in the EGCG@H, BPQDs@H, and control group. Cytocompatibility is key to wound healing as it is in direct contact with the wound tissue. Cell proliferation was also determined by MTT assay. The cell proliferation was sustainedly increased optical density (Fig. 5C). The proliferation of HUVECs after treatment of EGCG-BPQDs@H was significantly faster than other groups after 12- and 24-h incubation. The migration and proliferation among HUVECs indicated nanomaterials have good biocompatibility and the ability to improve wound healing, which could be attributed to the sustained release of EGCG, offering an appropriate microenvironment for distribution. Meanwhile, we evaluate the capacity of nanomaterials to promote epithelial by detecting the expression of basic fibroblast growth factor (bFGF) in HaCaT cells. It is widely accepted that bFGF.

is a kind of growth factor that takes a significant role in the therapy of skin trauma, neovascularization, and tissue regeneration [41]. Immunofluorescence observed on CLSM revealing expression of bFGF protein in HaCaTs was much higher in EGCG-BPQDs@H treatment (Fig. 5D).

Through western blot (Fig. 5E) and relative western blot quantitative analysis (Fig. 5F), enhanced expression of bFGF was discovered in EGCG-BPQDs@H group. Although the bFGF protein levels of other signal groups were higher than the control group, both were lower than the EGCG@BOQDs@H group, indicating the synergistic properties of black phosphorus quantum dots and EGCG. Therefore, the experimental results confirmed that the nanomaterials could accelerate epithelial healing by promoting the expression of bFGF in epithelial cells.

### EGCG-BPQDs@H facilitated vascularization

**Fig. 6:**
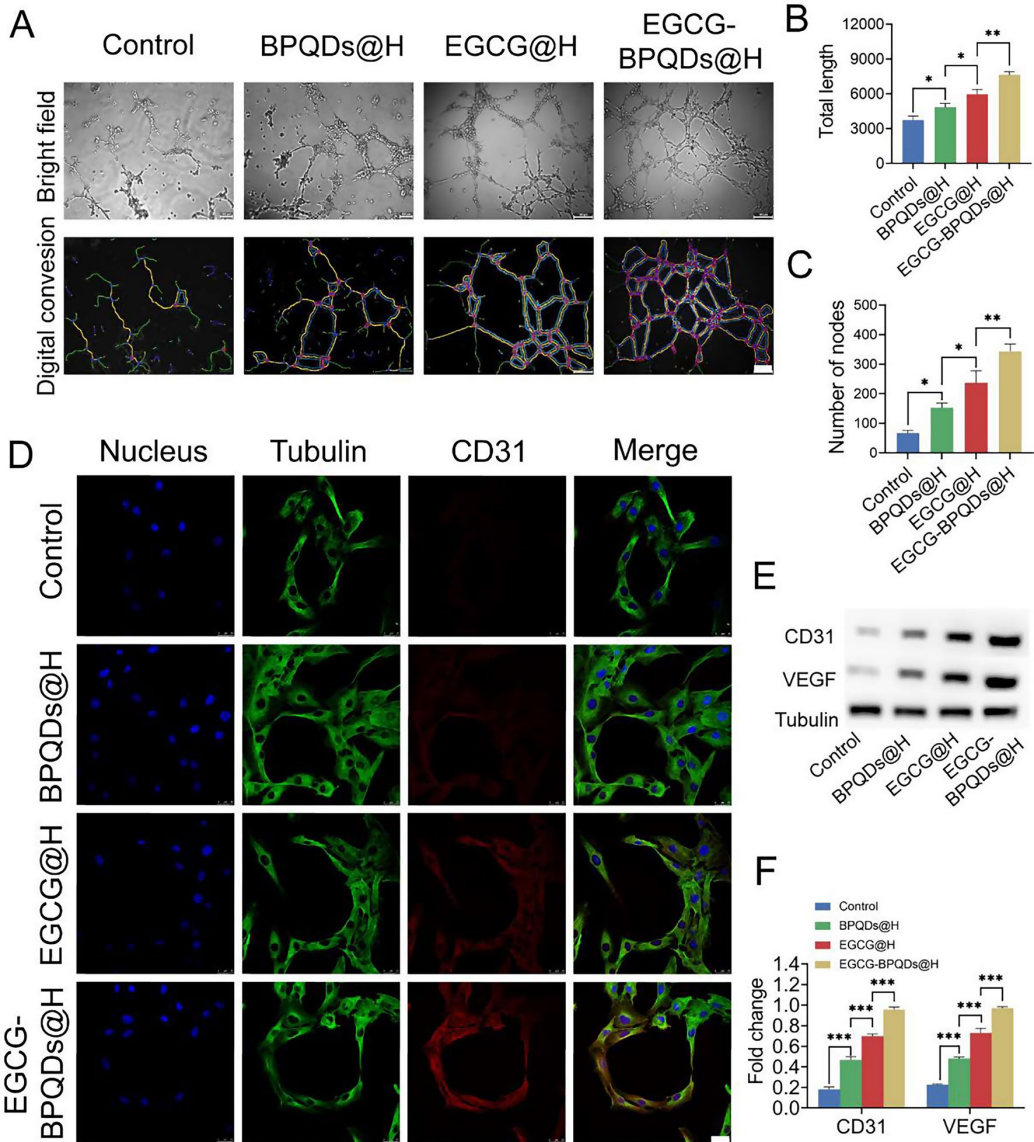
Angiogenic tubular formation and relative protein expression of endothelial cells in vitro. **A** Tubular formation on Matrigel and digital analysis conversion of HUVECs in different treatments, bar = 100 μm. (B, C) Total length and the number of nodes were quantified. **D** CLSM images of the expression analysis of CD31, bar = 25 μm. **E** The expression level of CD31 and VEGF protein. **F** Quantification analysis of the CD31 and VEGF protein. *P < 0.05, **P < 0.01, ***P < 0.001

Angiogenesis is developing new capillaries to form the original microvasculature, which is a crucial stage in wound healing [42]. Total processes involved the proliferation, migration, alignment, and germination of endothelial cells and connections between cells, tubular structures, and lumina [43]. We conducted the Matrigel experiment to simulate the angiogenesis of endothelial cells on connective tissue membrane. As shown in Fig. 6A, HUVECs incubated on the Matrigel after different materials treatments formed tube networks and mesh-like circles to a different degree. The HUVECs treated with EGCG@H and EGCG-BPQDs@H exhibited more notable angiogenesis indexes. Furthermore, longer total lengths, enhanced number of nodes were observed in EGCG-BPQDs@H treatment (Fig. 6B and C). These results identified that EGCG-BPQDs@H served as an ideal extracellular matrix to facilitate endothelial cell tubule formation in vitro, which may accelerate endothelization and angiogenesis in impaired tissues.

To further identify our inference, we performed CLSM and western blot to examine the expression of the angiogenesis-associated proteins. It has been widely demonstrated that CD31 and VEGF can assess the level of angiogenesis. The highly expressed CD31 indicates the tight junctions between endothelial cells and is involved in the formation of blood vessels [44]. EGCG@H exhibited a modicum improvement in the expression of CD31. By contrast, EGCG-BPQDs@H further enhanced the expression level of CD31 (Fig. 6D). Moreover, the result of western blot acted by immunofluorescence staining (Fig. 6E). The expression of CD31 in the group with the treatment of EGCG@BOQDs@H was remarkably improved compared to other groups (Fig. 6F). Undoubtedly, the expression of VEGF in EGCG-BPQDs@H treatment was higher than other groups. All these results suggested that we had proved once again that EGCG-BPQDs@H could ameliorate wound healing through promoting angiogenesis according to the relative protein expression. In combination with these findings, our study provides supporting evidence for using NIR-responsive, synergistic antimicrobial, sustained-release hydrogels of EGCG-modified BPQDs to promote MIDBW healing. To further investigate the healing effect in more detail, we carried out experiments in vivo.

### In vivo assessment of burn-wound healing

**Fig. 7:**
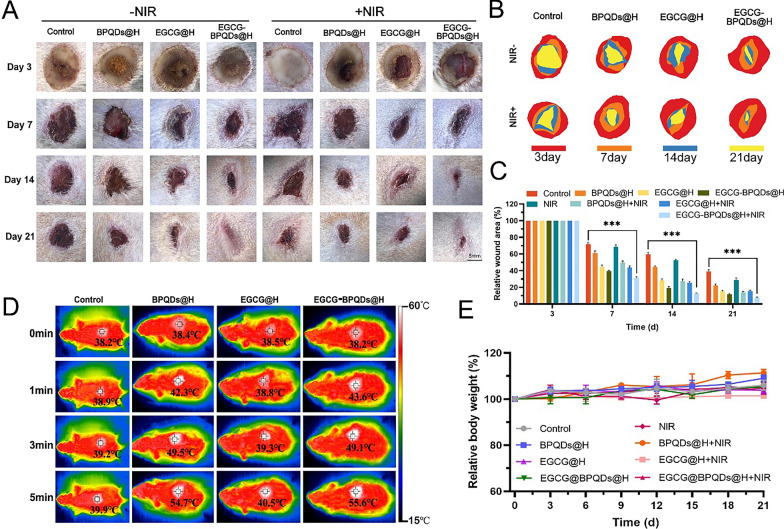
Evaluation effects of accelerating diabetic infected burn-wound in vivo. **A** Images of infected burn-wound healing at different times, bar = 5 mm. **B** Traces of wound-bed closure during 21 days for each treatment. **C** Corresponding statistical graph of relative wound area from each group with different treatments. **D** Photothermal images of rats treated with hydrogel, BPQDs@H, EGCG@H, and EGCG-BPQDs@H with NIR irradiation at different times. **E** Changes in body weight of the rats

To further explore the therapeutic benefits of EGCG-BPQDs@H in burn-wound healing in vivo, the digital photographs of burn-wound showed male Sprague–Dawley diabetic rats infected burning wounds (Fig. 7A). The burn-wounds of diabetic rats treated with EGCG-BPQDs@H with or without NIR revealed accelerated wound closure in the continued bacterial infected burn-wound healing experiment (Fig. 7B). More specifically, the relative wound area on day 21 exhibited that local EGCG-BPQDs@H with NIR irradiation application leading to 92.4% wound closure, which was conspicuously higher than the control group (61.1%) (Fig. 7C). Remarkably, the elevated temperature induced by the photothermal effect was detected upon NIR irradiation. The highest temperature of wound region treated with BPQDs@H or EGCG-BPQDs@H was ~ 55℃ after 5 min’ irradiation, revealing prominent photothermal performance of black phosphorus quantum dots (Fig. 7D).

One major concern about photothermal therapy (PTT) is that local hyperpyrexia may cause secondary injury to the burn wound. The impairment to cells around the wound caused by PTT is far less serious than that caused by bacterial infection. This is acceptable for PTT-mediated improvement of blood flow and increased oxygen supply to promote burn-wound healing. In contrast, the trauma area in the control or EGCG@H groups showed temperature increase of only 1.7 ℃ and 2.0 ℃. Moreover, no significant body weight fluctuations were observed in all groups during the treatment period (Fig. 7E), indicating the satisfactory bio-safety of these tested nanomaterials. In the meantime, an obvious healing effect can also be observed in the treatment of EGCG-BPQDs@H compared with other single treatment groups. While ECGC@BPQDs@H + NIR group indicating the minimum wound area and fastest healing rate, which revealed the EGCG-BPQDs@H could be used to apply the drug-resistant bacterial infected diabetic wound in vivo with assistance of the photothermal and photocatalytic effects. Next, to verify the in vivo antimicrobial activity, we collected MRSA from the wound skin and performed a bacterial colony count test using the LB agar plate smear method. As shown in Additional file 1: Fig. S6A, few colonies were seen in the EGCG-BPQDs@H + NIR group, and the results were consistent with in vitro experiments. For the clinical treatment of diabetic burn patients, this near-infrared light-responsive composite hydrogel can promote repair and resistance to drug-resistant bacterial infections. In addition, the superficial application and the ability to achieve rapid wound healing provide ease and feasibility for clinical application. Phototherapy has attracted a great deal of research due to its remote controllability, deep tissue penetration, and non-invasive nature [45–48]. Its potential for a wide range of biological applications includes, but is not limited to, trauma repair, tumor treatment [49–51], and probe imaging [52, 53]. As its antibacterial mechanism is different from that of antibiotics, the use of PTT can effectively avoid the development of resistance. These are very attractive for clinical applications. And by H&E staining (Additional file 1: Fig. S6B), many inflammatory cells were found in the wounds treated with the control group. After treatment with EGCG-BPQDs@H with NIR, there was a significant reduction in the number of inflammatory cells in the wounds relative to the other groups.

**Fig. 8:**
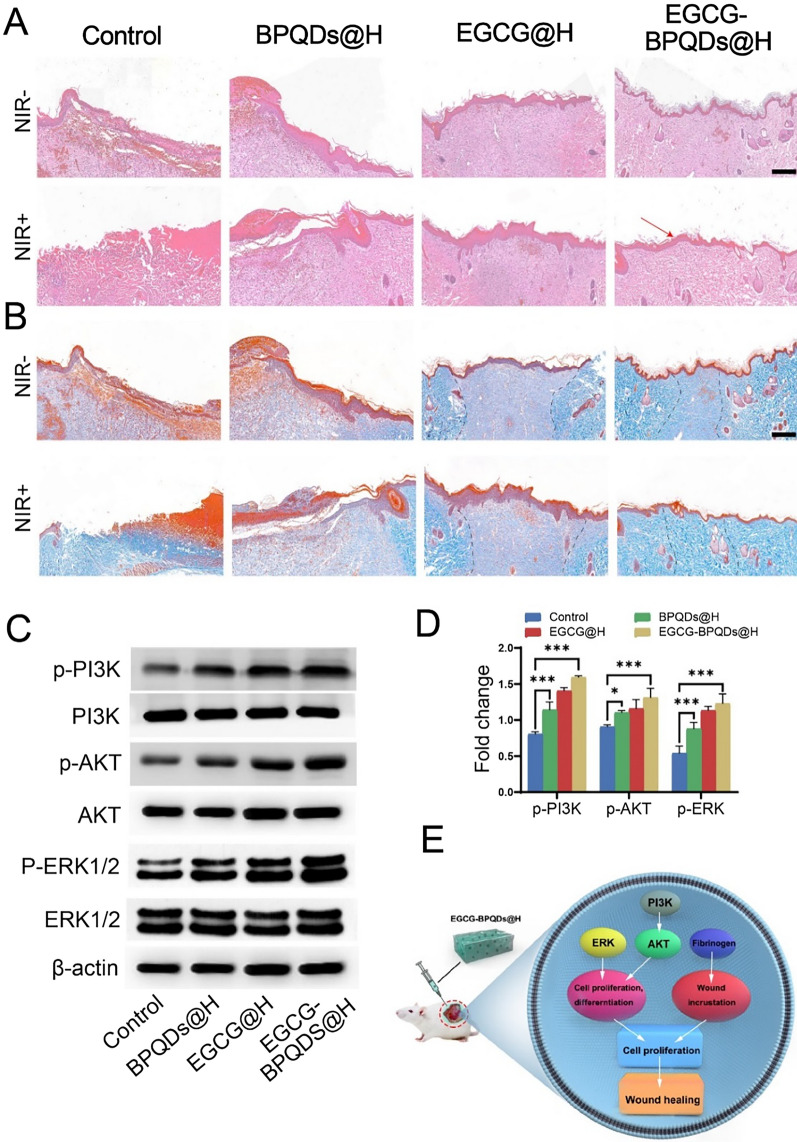
Evaluation on the healing-promoting effect of the nanoplatforms on infected burns rats. **A** H&E staining of wound sites with different treatments, the red arrow indicates intact epidermis, bar = 200 μm. **B** Masson staining of the wound tissues, dotted line indicates collagen at the wound, bar = 200 μm. **C** Western blot analysis. **D** Quantification for the molecules involved in the signaling pathways for burn wound healing. **E** Scheme diagram showing nanomaterials direct cell proliferation and enhanced fibrinogen expression to accelerate wound healing

To observe the histological changes in wound healing, H&E and Masson staining were performed to study the mechanism behind this process. Compared with other groups, control and BPQDs@H groups could not develop intact epidermal tissue. EGCG@H and EGCG-BPQDs@H groups had completed the procedure of re-epithelialization. The epithelium of the EGCG-BPQDs@H with NIR group was smoother and more regulated than of the EGCG-BPQDs@H group, and follicles began to develop around the wound (Fig. 8A). Matson staining was then applied to samples from different groupings to reflect collagen deposition in the sarcomeres. As a result, only a few collagen fiber bundles were formed in the control and BPQDs@H groups. There was still large spaced and a loose reticular arrangement between the collagen fibers and skin tissue remaining. In contrast, EGCG-BPQDs@H group had an intact epidermal structure and dense, regular collagen fibers with a larger collagen deposition area at the wound (Fig. 8B). Subsequently, western blot analysis revealed that EGCG-modified BPQDs had increased the expression of key signaling molecules in the PI3K/AKT and ERK1/2pathways (Fig. 8C). Quantitative analysis revealed that phosphoinositide 3-kinase (PI3K), protein kinase B (AKT), and extracellular signal-regulated kinase (ERK1/2) were significantly increased in the EGCG-BPQDs@H group compared to the control group (Fig. 8D). These signaling pathways direct cells proliferation and differentiation, which was attributed to the stimulative effect of the phosphate/phosphonate ions following the degradation of BPQDs. Figure 8E summarizes the previous findings on how nanomaterials induce signaling pathways to direct cell proliferation and differentiation. In addition, we subcutaneously injected the hybrid hydrogel into the dorsal side of the rats (Additional file 1: Fig. S7). The experimental results revealed a gradual decrease in hydrogel content due to in vivo metabolism. During this process, there was no swelling or necrosis of the tissue around the hydrogel, indicating that the hydrogel system has favorable biocompatibility.

### Biological safety in vivo

**Fig. 9:**
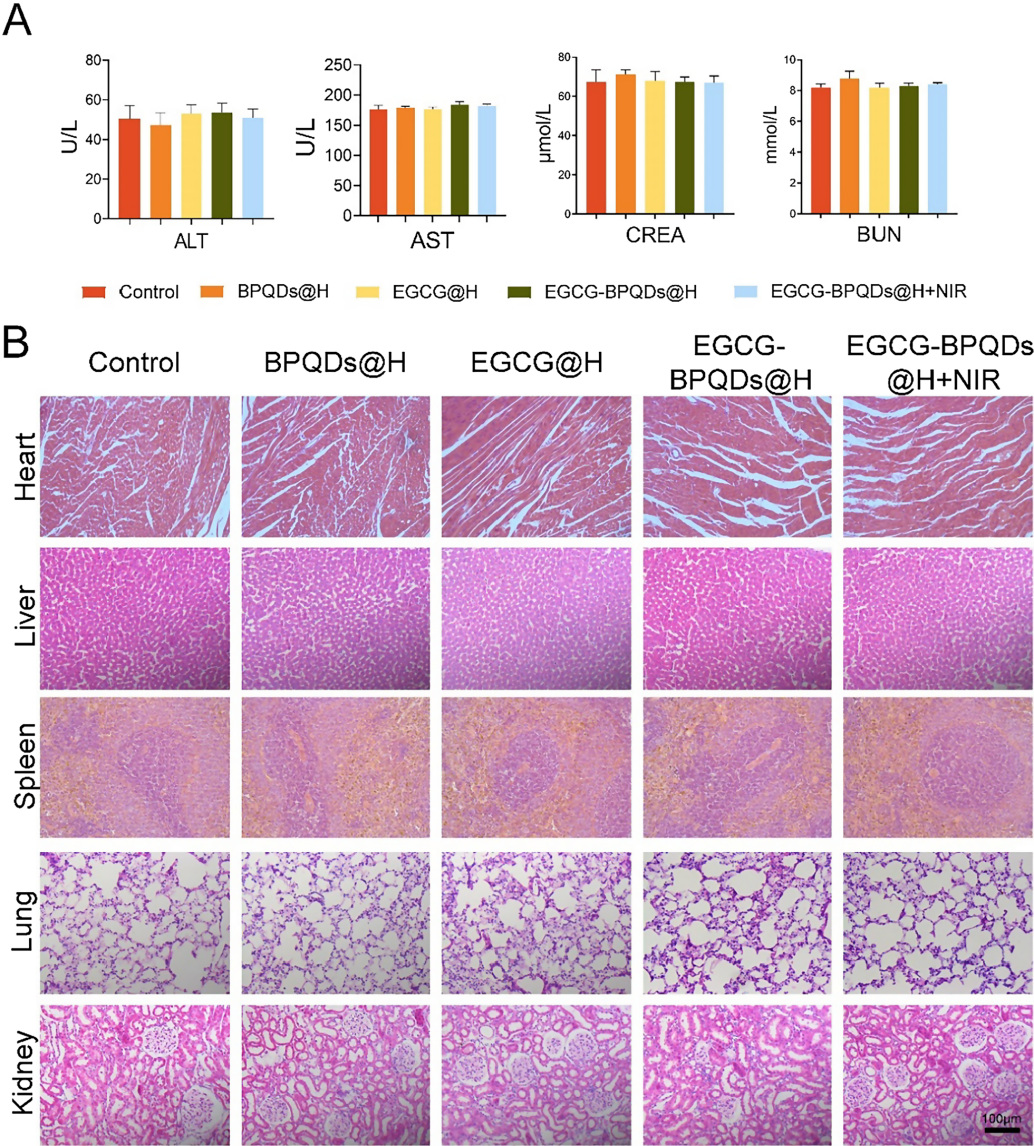
Preliminary toxicity study in vivo. **A** Hepatorenal function test for ALT, AST, CREA and BUN. **B** Histological toxicological observation of H&E staining of the heart, liver, spleen, lung, and kidney with different treatments, bar = 100 μm

The results presented above fully attested to the prominent properties and potential of EGCG-BPQDs@H with NIR irradiation treatment in antibacterial activity and wound healing. Meanwhile, it is crucial to assess the biocompatibility of combined photothermal therapy in vivo for its practical application. Due to the characteristics of nanomaterials in direct contact with the burn-wound, blood biochemical analysis, and histological examination were applied to evaluate the biocompatibility of the cooperative treatment in vivo. BP has been widely used in drug delivery with high biocompatibility [54]. Additionally, biodegradation products of BP are phosphate ions and phosphonates, which are normally present in the blood [55]. Biochemistry analyses were carried out for blood at the twenty-first day. The liver function indexes (such as ALT and AST) and kidney function indexes (such as BUN and CREA) in the EGCG-BPQDs@H + NIR system were in the normal range (Fig. 9A). Subsequently, for EGCG-BPQDs@H + NIR system, H&E staining did not detect apparent inflammation and necrosis in the normal anatomical structure of various organs after treatment (Fig. 9B). In addition, we co-cultured the nanomaterial hydrogel with HaCaTs and found that the cells had good morphology with no obvious deformation or necrosis, proving their good biocompatibility (Additional file 1: Fig. S8). The hemolytic properties of the nanomaterial hydrogels were evaluated by measuring the hemolytic properties of red blood cells. The hemolysis rate of BPQDs@H, EGCG@H, and EGCG-BPQDs@H was significantly lower (less than 5%) after incubation of erythrocytes with each group of composites compared with water, indicating that the composite hydrogels had no significant hemolytic effect (Additional file 1: Fig. S9). These results strongly demonstrated that the EGCG-BPQDs@H with NIR irradiation system could be competent for a safe and effective therapeutic strategy to accelerate infected burn-wound healing.

## Conclusion

In summary, the present work successfully constructed photocatalytic, photothermal effects of EGCG modified black phosphorus quantum dots as multifunctional nanoplatforms and evaluated their antibacterial ability and wound healing activity in MRSA-infected wounds under diabetic conditions. The resulted EGCG-BPQDs present an effective sterilization rate of 88.6% for MRSA. Combining the photothermal effect of our sterilization depot promotes the release of EGCG, and EGCG-BPQDs@H could produce ^1^O_2_ in the case of NIR light. Molecular biology analysis demonstrated that EGCG-BPQDs significantly upregulated CD31 nearly fourfold and basic fibroblast growth factor (bFGF) nearly twofold, which were beneficial for promoting the proliferation of vascular endothelial cells and skin epidermal cells. Under NIR irradiation, EGCG-BPQDs hydrogel (EGCG-BPQDs@H) treated MIDBW area could rapidly raise temperature up to 55 °C for sterilization. The MIBDW closure rate of rats after 21 days of treatment was 92.4%, much better than 61.1% of the control group. The engineered EGCG-BPQDs were found to promote MIDBW healing by triggering the PI3K/AKT and ERK1/2 signaling pathways, enhancing cell proliferation and differentiation. In addition, the intravenous circulation experiment showed good biocompatibility of EGCG-BPQDs. No obvious damage to major rat organs was observed. The obtained results demonstrate that EGCG-BPQDs promise multifunctional nanoplatforms for MIDBW healing. The treatment of acute and chronic difficult-to-heal skin wounds associated with infection has been an important clinical issue. For the clinical treatment of diabetic burn patients, this NIR light-responsive composite hydrogel can promote repair and resistance to drug-resistant bacterial infections. In addition to the superficial application and the ability to achieve rapid wound healing also provide convenience and feasibility in clinical applications. Thus, the results of this study provide strong evidence for the clinical applicability of EGCG-BPQDs@H for the successful treatment of wounds in complex disease conditions such as diabetes with infected burn wounds. This type of hydrogel dressing is expected to provide clinical candidates in the future.

## Supplementary Information

**Additional file 1.** Additional figures.

## Data Availability

The datasets used and/or analyzed during the current study are available from the corresponding author on reasonable request.

## Abbreviations

MRSA: Methicillin-resistant Staphylococcus aureus
EGCG: Epigallocatechin gallate
BPQDs: Black phosphorus quantum dots
NIR: Near-infrared
bFGF: Basic fibroblast growth factor
BP: Black phosphorus
^1^O_2_: Singlet oxygen
*E. coli*: Gram-negative bacteria Escherichia coli
DMEM: Dulbecco's modified Eagle's medium
MTT: 3-(4,5-Dimethylthiazol-2-yl)-2,5-diphenyl tetrazolium bromide
FBS: Fetal bovine serum
HaCat: Human skin keratinocytes cells
HUVEC: Human umbilical vein endothelial cells
ABDA: The 2,2’-bis(anthracene-9,10-diylbis(methylene))-dimalonic acid
NMP: 1-Methyl-2-pyrrolidone
DLS: Dynamic laser scattering
TEM: Transmission electron microscope
CLSM: Confocal laser scanning microscopy
STZ: Streptozotocin
SEM: Scanning electron microscope
DCFH-DA: 2′,7′-Dichlorodihydrofluorescein diacetate
DAPI: 4′,6-Diamidino-2-phenylindole solution
DMSO: Dimethyl sulfoxide
ALT: Alanine aminotransferase
AST: Aspartate aminotransferase
CREA: Creatinine
