# Correction to: Tea polyphenol modified, photothermal responsive and ROS generative black phosphorus quantum dots as nanoplatforms for promoting MRSA infected wounds healing in diabetic rats

**DOI:** 10.1186/s12951-022-01408-7

**Published:** 2022-04-19

**Authors:** Shibo Xu, Linna Chang, Yanan Hu, Xingjun Zhao, Shuocheng Huang, Zhenhua Chen, Xiuli Ren, Xifan Mei

**Affiliations:** Jinzhou Medical University, JinzhouLiaoning, 121001 China

## Correction to: J Nanobiotechnol (2021) 19:362 https://doi.org/10.1186/s12951-021-01106-w

Following the publication of the original article [1], the authors reported that Fig. 8A was incorrect. The corrected Fig. 8 and the figure caption are given. The corrections do not affect the results and conclusions. All authors agree to these corrections and apologize for this error.

**Fig. 8:**
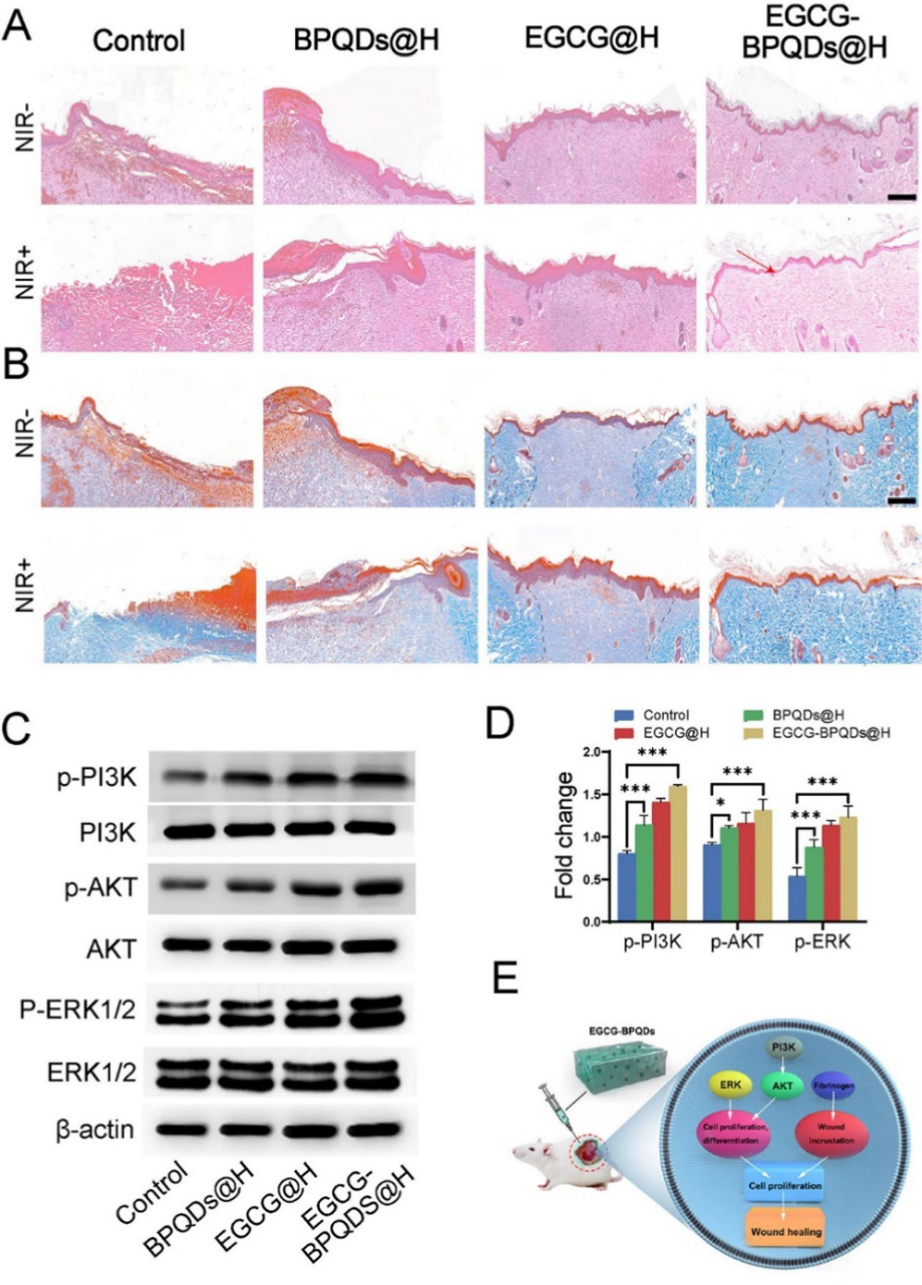
Evaluation on the healing-promoting effect of the nanoplatforms on infected burns rats. **A** H&E staining of wound sites with different treatments, the red arrow indicates intact epidermis, bar = 200 μm. **B** Masson staining of the wound tissues, dotted line indicates collagen at the wound, bar = 200 μm. **C** Western blot analysis. **D** Quantification for the molecules involved in the signaling pathways for burn wound healing. **E** Scheme diagram showing nanomaterials direct cell proliferation and enhanced fibrinogen expression to accelerate wound healing
