# Supplementary material for: Tea polyphenol modified, photothermal responsive and ROS generative black phosphorus quantum dots as nanoplatforms for promoting MRSA infected wounds healing in diabetic rats
Source: J Nanobiotechnology. 2021 Nov 10;19:362. doi: 10.1186/s12951-021-01106-w (PMC8579683; doi:10.1186/s12951-021-01106-w)
Supplement: Supplementary file 1 — Additional file 1. Additional figures. [file 12951_2021_1106_MOESM1_ESM.docx]

**Additional Information**

Calculation of the photothermal conversion efficiency

According to previous reports[1,2], we calculated the photothermal conversion efficiency of the composite hydrogel according to the following formula:

$$\eta=\frac{hS\left( \Delta T_{max,mix}-\Delta T_{\max,H_{2}O} \right)}{I\left( 1-{10}^{-A_{808}} \right)}$$

(1)

$$hS=\sum mC_{p}/\tau_{s}$$

(2)

$$\tau_{s}=-t/ln\theta$$

(3)

$$\theta=\frac{T-T_{surr}}{T_{max}-T_{surr}}$$

(4)

where $h$ is the heat transfer coefficient, $S$ is the surface area of the container. $\tau_{s}$ is the sample system time constant. It should be noted that the mass ratio of polyvinyl alcohol in the composite hydrogel is only 8% and the water is completely encapsulated in the resultant hydrogel, so the specific heat capacity of the hydrogel can be close to that of pure water. In general, water is much hotter than other substances. Therefore, we consider only the mass and specific heat of water. $\Delta T_{max,mix}$and$\Delta T_{\max,H_{2}O}$are the maximum temperature change of composite hydrogel and water, respectively ($\Delta T_{max,mix}=38.4℃ ,\Delta T_{\max,H_{2}O}=1.7℃$). *I* is 6.275 W where the area of light spot is 2.51 cm^2^. A_808_ is the absorbance of composite hydrogel at the excitation wavelength of 808 nm (A_808_ = 8.74), *m* is the mass of water (m = 3 g), C*_p_* is specific heat capacity of water (CH_2_O = 4.2 J/(g**∙**℃)), and the value of $\tau_{s}$ is 157.8 s obtained from Fig. S2. *T* and *T*_surr_ are the solution temperature and the ambient temperature, respectively. According to Formula (2), we can get hS equals 0.0798, substituting into formula (1), η=46.7% of EGCG-BPQDs@H can be calculated.


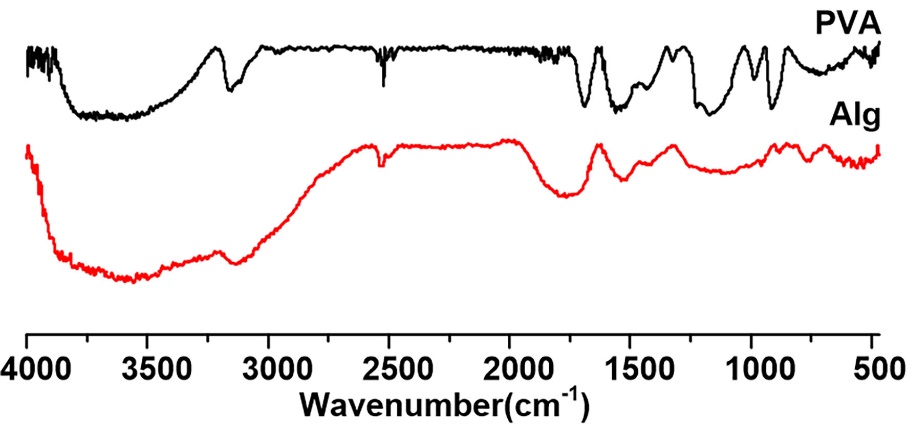


**Fig. S1.** FTIR spectra of PVA and Alg.


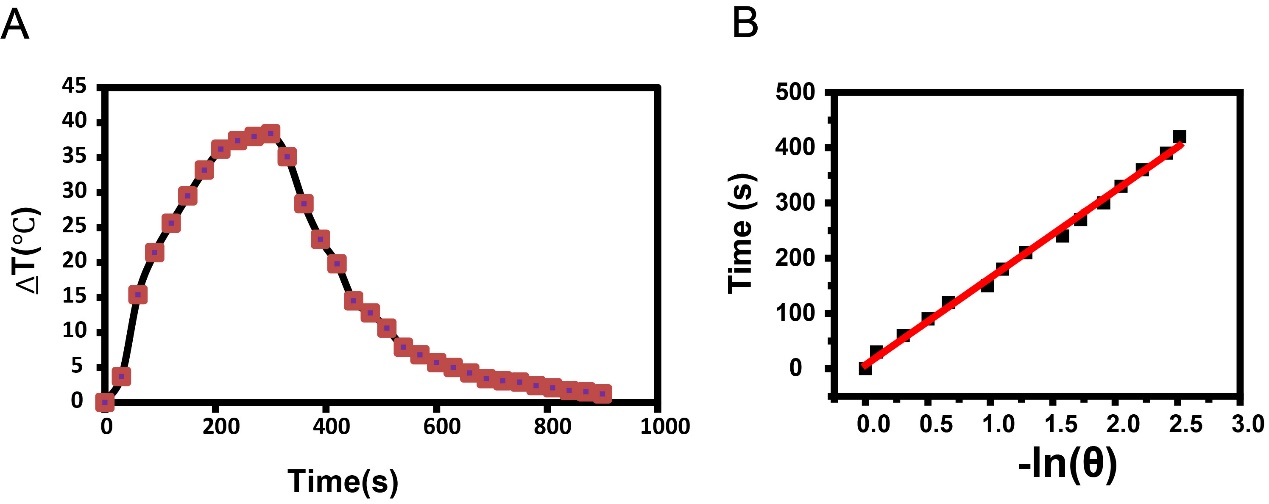


**Fig. S2.** (A) The photothermal response of EGCG-BPQDs@H in water with laser irradiation (808 nm, 2.5 W/cm^2^, 6 min) and then the laser was shut off. (B) Linear time data versus -ln (θ) obtained from the cooling period of NIR laser off.


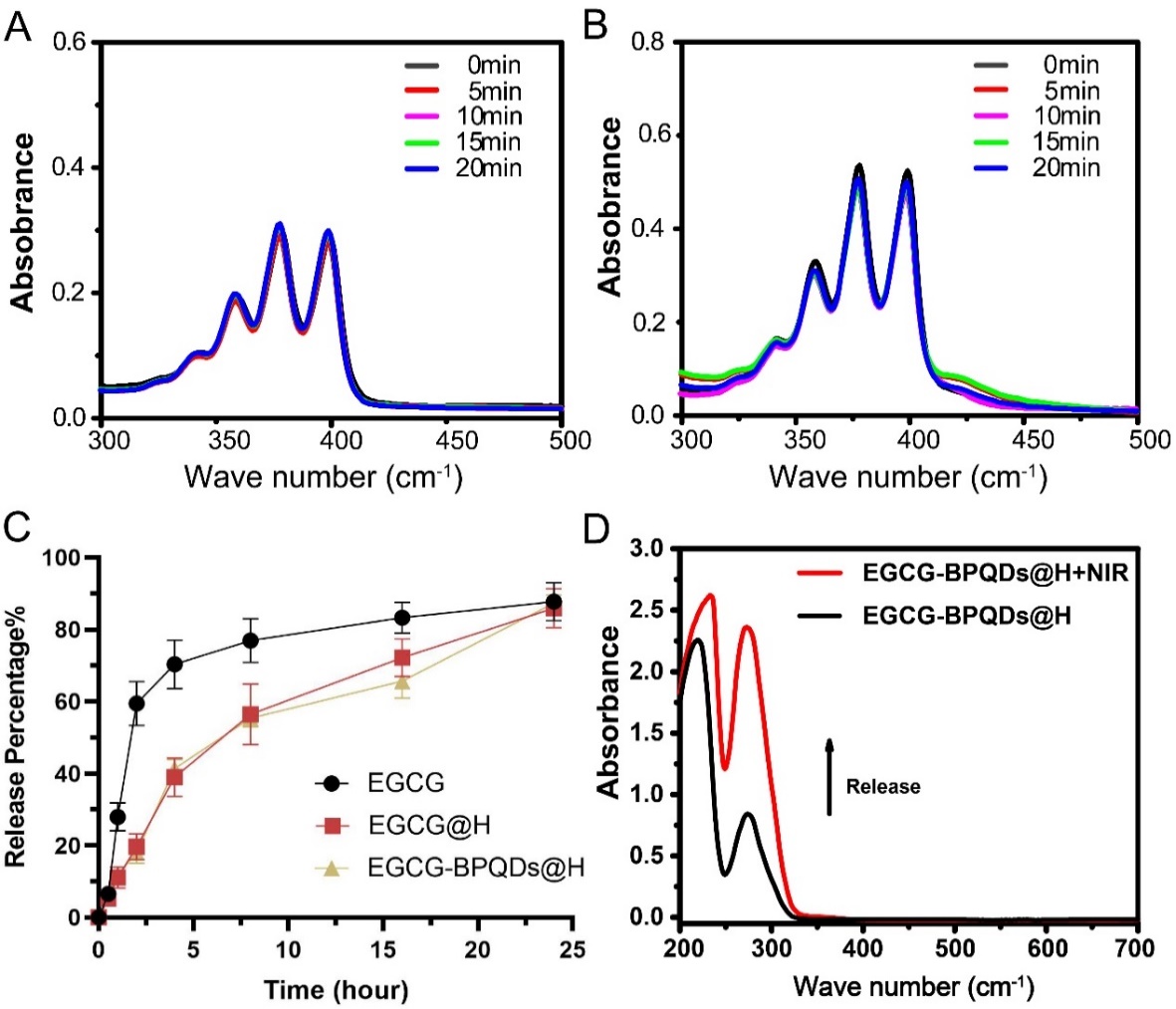


**Fig. S3.** Changes in the UV-vis absorption spectra of ABDA solution (A) and ABDA solution containing EGCG (B) after irradiation with NIR laser for different times. (C) In vitro release profile of EGCG, EGCG@H and EGCG-BPQDs@H over 24 hours. Data are expressed as means ± SD (n = 3). (D) UV-vis absorbance of samples after 5 minutes of NIR irradiation (2.5 W/cm^2^, 808 nm).


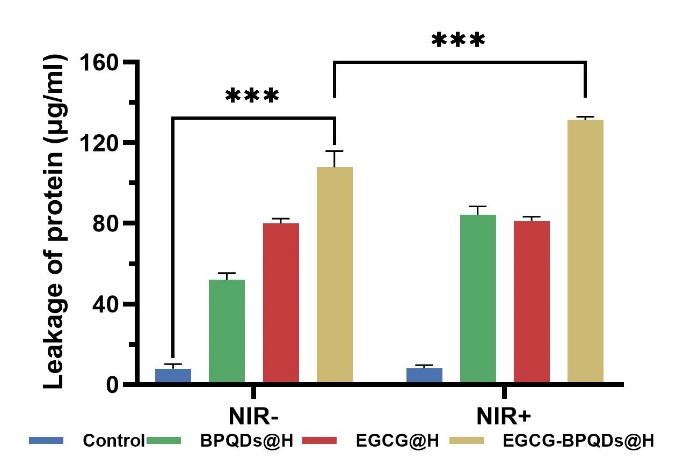


**Fig. S4.** Protein leakage of MRSA suspensions treated with different materials under NIR irradiation or not.


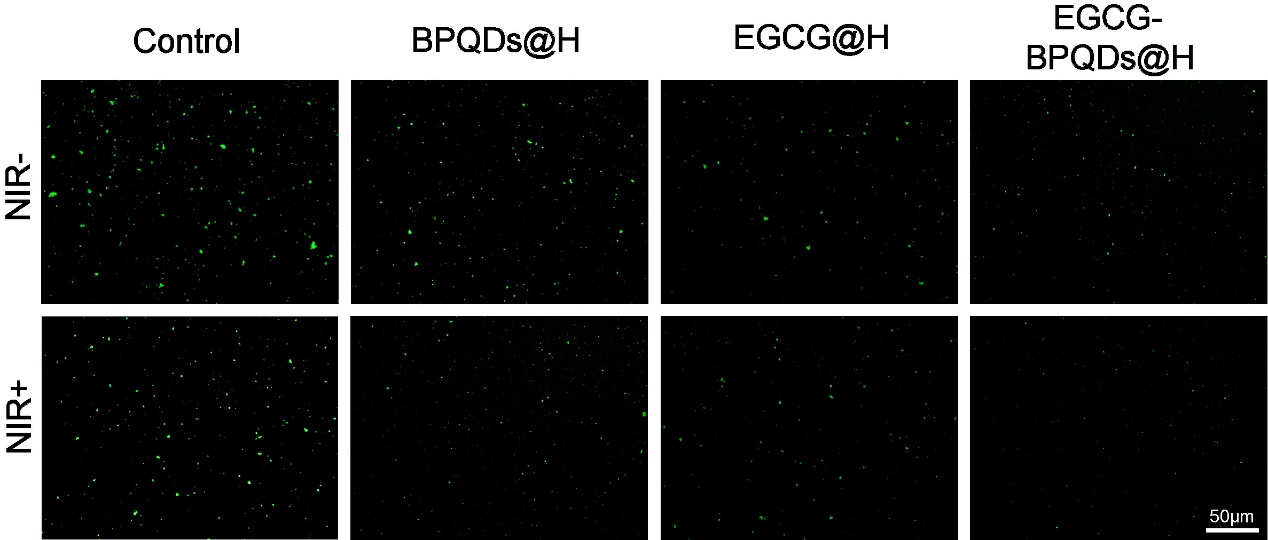


**Fig. S5.** Fluorescent staining of bacterial biofilms, bar=50μm.


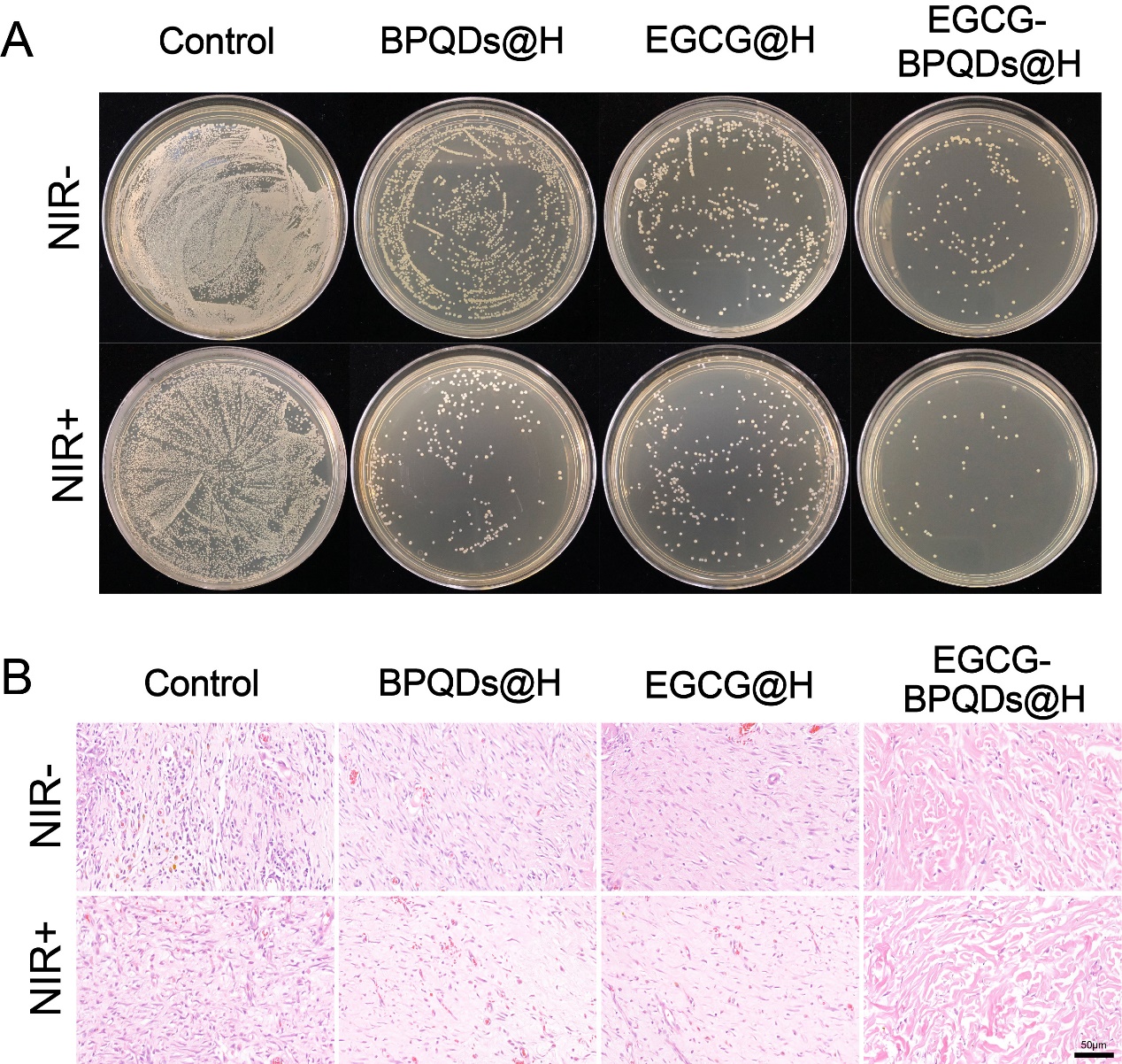


**Fig. S6.** EGCG-BPQDs@H reduces bacterial colonization of diabetic wounds and inhibits inflammation. (A) Amount of remaining viable bacteria in tissue homogenate after treatment

(B) H&E staining for infiltration of inflammatory cells in tissues, bar=50μm.


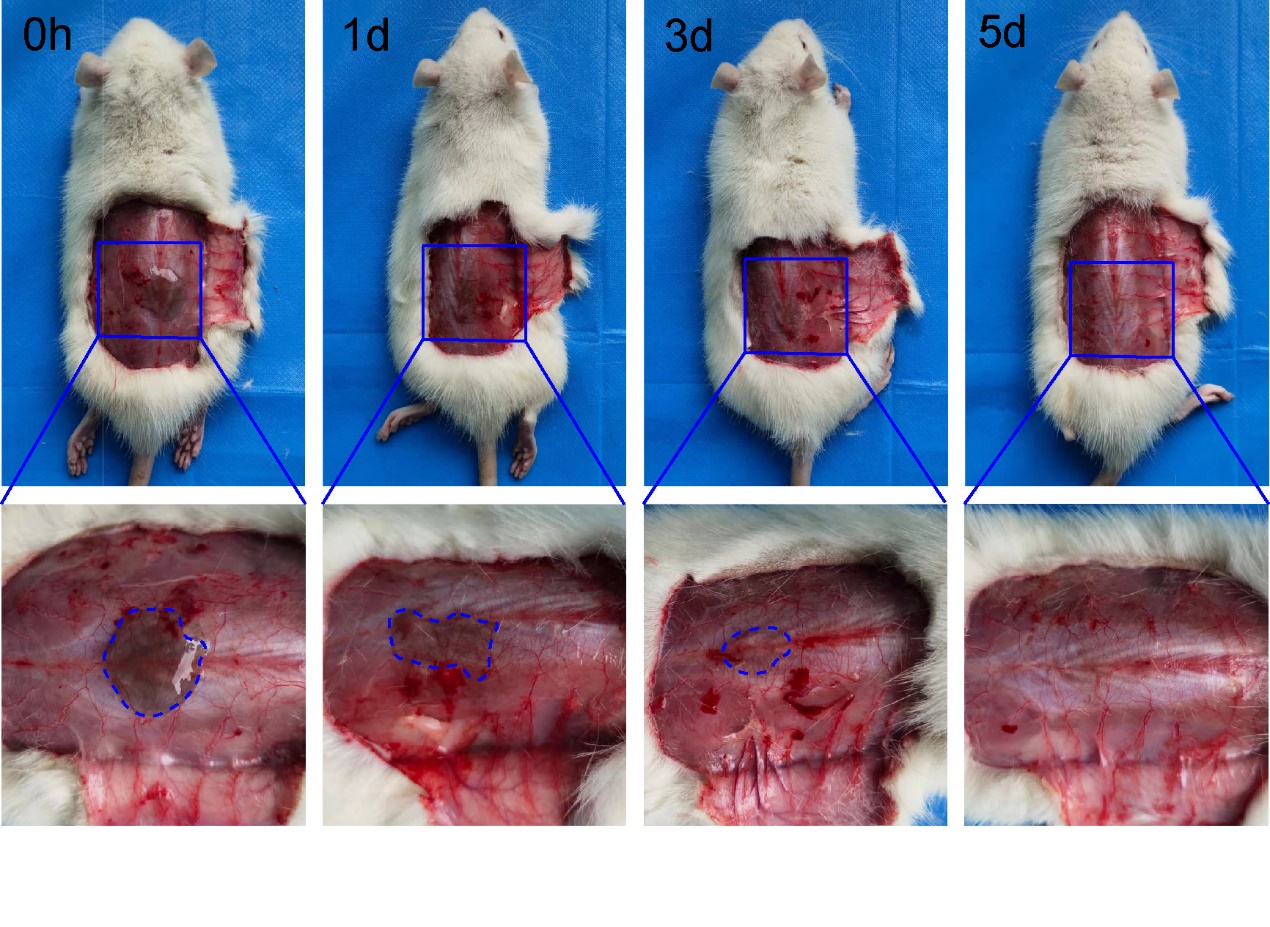


**Fig. S7.** Gross in vivo degradation of composite hydrogels at different times.


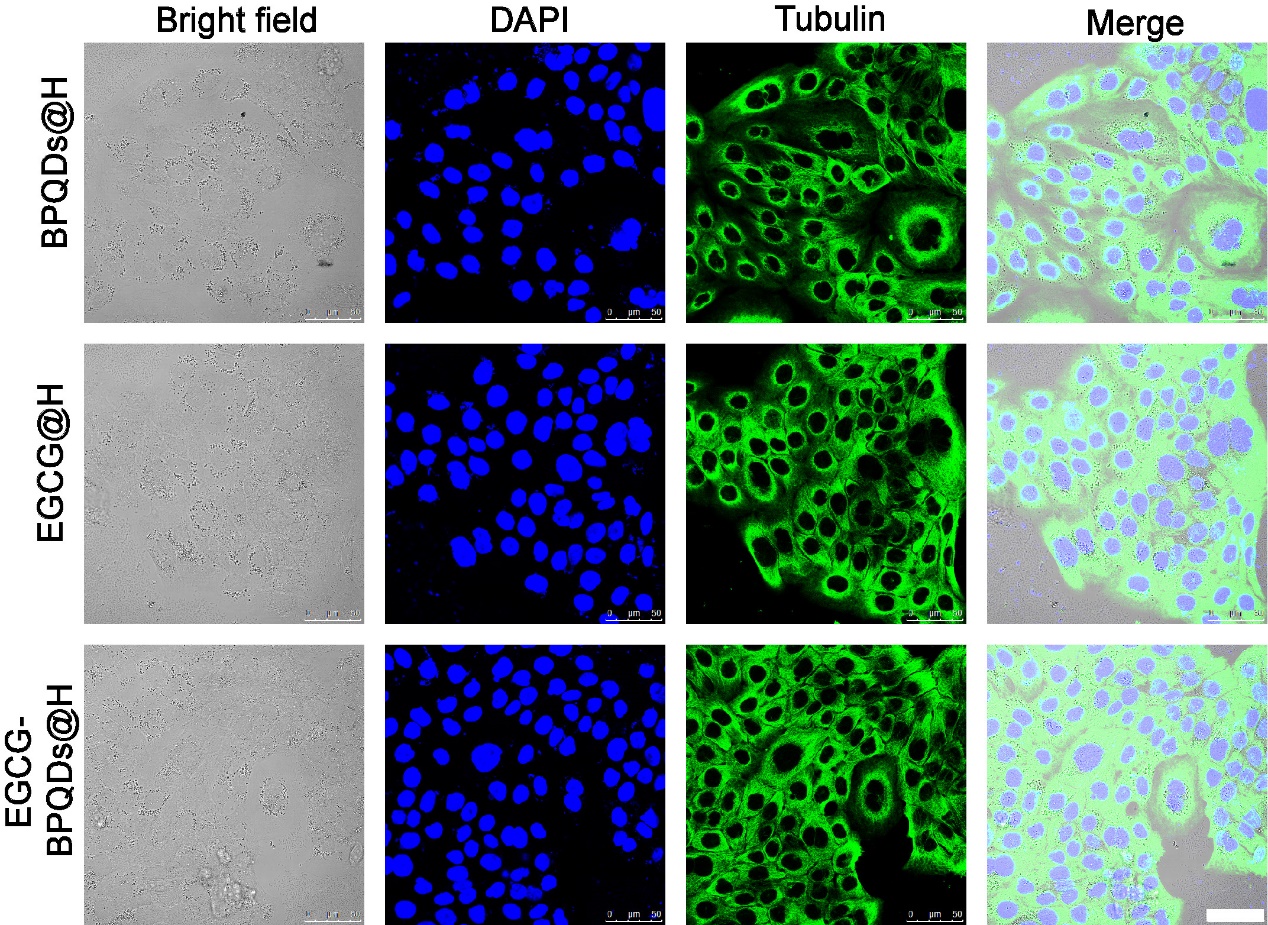


**Fig. S8.** Confocal microscope fluorescence photographs of composite nanomaterial hydrogels co-cultured with HaCaTs, bar=25μm.


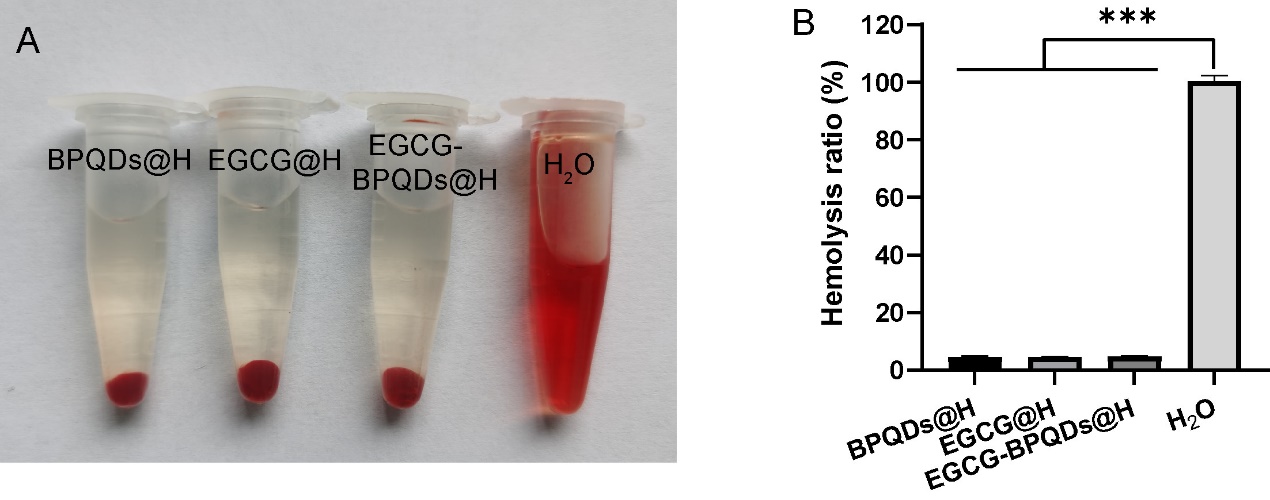


**Fig. S9.** Hemocompatibility of composite hydrogels and the corresponding scores. (A) picture of erythrocyte after incubating with composite hydrogels. H_2_O were used as control. (B) Hemolysis ratio in corresponding groups.

References

[1] Yang G, Wan X, Gu Z, et al. Near infrared photothermal-responsive poly (vinyl alcohol)/black phosphorus composite hydrogels with excellent on-demand drug release capacity. Journal of Materials Chemistry B, 2018, 6(11): 1622-1632.

[2] Zheng Y, Liang Y, Zhang D, et al. Fabrication of injectable CuS nanocomposite hydrogels based on UCST-type polysaccharides for NIR-triggered chemo-photothermal therapy. Chemical Communications, 2018, 54(98): 13805-13808.
